# Compositional discordance between prokaryotic plasmids and host chromosomes

**DOI:** 10.1186/1471-2164-7-26

**Published:** 2006-02-15

**Authors:** Mark WJ van Passel, Aldert Bart, Angela CM Luyf, Antoine HC van Kampen, Arie van der Ende

**Affiliations:** 1 Academic Medical Center, Department of Medical Microbiology, Amsterdam, The Netherlands; 2 Academic Medical Center, Bioinformatics Laboratory, Amsterdam, The Netherlands

## Abstract

**Background:**

Most plasmids depend on the host replication machinery and possess partitioning genes. These properties confine plasmids to a limited range of hosts, yielding a close and presumably stable relationship between plasmid and host. Hence, it is anticipated that due to amelioration the dinucleotide composition of plasmids is similar to that of the genome of their hosts. However, plasmids are also thought to play a major role in horizontal gene transfer and thus are frequently exchanged between hosts, suggesting dinucleotide composition dissimilarity between plasmid and host genome. We compared the dinucleotide composition of a large collection of plasmids with that of their host genomes to shed more light on this enigma.

**Results:**

The dinucleotide frequency, coined the genome signature, facilitates the identification of putative horizontally transferred DNA in complete genome sequences, since it was found to be typical for a certain genome, and similar between related species. By comparison of the genome signature of 230 plasmid sequences with that of the genome of each respective host, we found that in general the genome signature of plasmids is dissimilar from that of their host genome.

**Conclusion:**

Our results show that the genome signature of plasmids does not resemble that of their host genome. This indicates either absence of amelioration or a less stable relationship between plasmids and their host. We propose an indiscriminate lifestyle for plasmids preserving the genome signature discordance between these episomes and host chromosomes.

## Background

Prokaryotic mobile elements such as plasmids play key roles in biological research as molecular biological vectors. More importantly, they have contributed substantially to genome evolution throughout biological history [1]. In addition, various studies have demonstrated the importance of horizontal transfer of genes via mobile elements, for example in virulence [2], adaptation [3] and most well-known in conferring antibiotic resistance [4].

The genome signature, which is the set of dinucleotide relative abundance values [5], is one of the parameters available to identify putative horizontally transferred DNA. The genome signature is typical for a given bacterial genome and similar between closely related genomes. These host-specific patterns are thought to result from differences in the replication and/or mismatch repair systems between species [6]. Due to its species-specific nature, this signature enables easy detection of anomalous genomic regions [7]. Recently, we developed an application based on the genome signature that allows the comparison of the genome signature of a sequence as small as 1 kbp with that of a sequenced genome [8,9].

Most plasmids depend on the host replication machinery and possess partitioning genes. These properties confine plasmids to a limited number of hosts, yielding a close and presumably stable relationship between plasmid and host. Genome signature compatibility between a plasmid and its host could indicate a long-term association, for example via strict vertical transmission, whereas high genomic dissimilarity scores between the plasmid and the host could indicate separate evolutionary histories. Although Wong and co-workers have previously suggested that plasmids are more dissimilar from chromosomes than chromosomes from the same strain amongst each other, the extent of their analysis was limited [10]. We therefore analyzed genome signature dissimilarities of 230 plasmid sequences with representative host chromosome sequences.

## Results

### Sequence length independence genome signature comparison between a plasmid and the genome of its host

**Figure 1:**
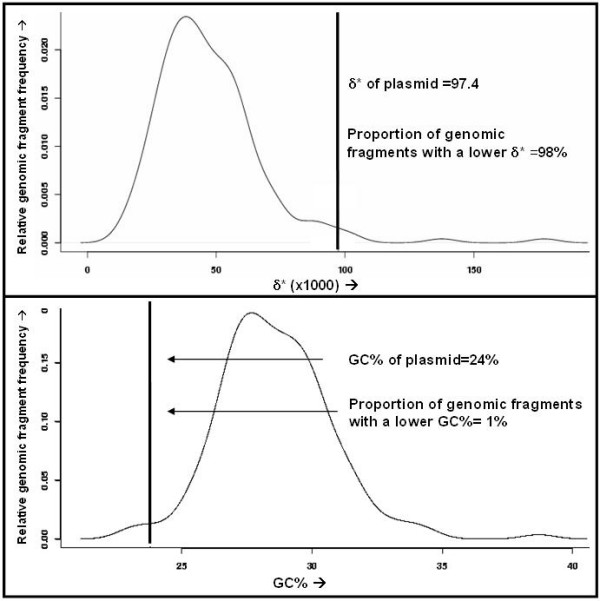
Comparison of the δ* value and the GC content of plasmid lp5 with the δ* value and the GC content of the genome of *B. burgdorferi *B31. The chromosome sequence (here *B. burgdorferi *B31) is divided in non-overlapping fragments with a size equal to the length of the input plasmid sequence (the Borrelia plasmid lp5, NC_000957), after which a frequency distribution is made for both δ* and the GC percentage scores. The δ* value of the input plasmid sequence is plotted vertically in the fragment distribution, indicating the proportion of genomic fragments with a lower δ* value. Consequently, this analysis can be performed for the GC content. The value of the plasmid GC content plotted in the fragment distribution indicates the proportion of genomic fragments with a lower GC percentage.

Genome signature dissimilarity scores (δ*) are calculated as described previously [8,11], with δ* being the average absolute dinucleotide relative abundance difference (see methods). For this analysis, the relevant chromosome sequence, in Fig. 1 that of *Borrelia burgdorferi *B31, is divided in non-overlapping fragments of identical length as the *B. burgdorferi *B31 plasmid lp5. The distribution of the δ* scores between these genomic fragments and the host genome sequence are visualised in a frequency distribution plot, with the δ* between plasmid and host indicated as a vertical line (Fig. 1). For plasmid lp5 we find a high δ* value of 97.4, and from the position of this δ* value in the distribution it is deduced that 98% of the *B. burgdorferi *B31 chromosomal fragments have a lower δ* value than that of plasmid lp5 (Fig. 1A). A similar procedure to compare the GC content of plasmid lp5 to that of the chromosome indicates that only 1% of the chromosomal fragments have a lower GC content than plasmid lp5 (Fig. 1). These results indicate a substantial compositional difference between plasmid lp5 and the genome of *B. burgdorferi *B31 This approach allows us to compare the genome signature differences and GC content deviations between different plasmid/host genomic fragment combinations from entries of the Plasmid Genome Database [12].

### Genome signature comparison between plasmids and the sequenced genome of their host

**Figure 2:**
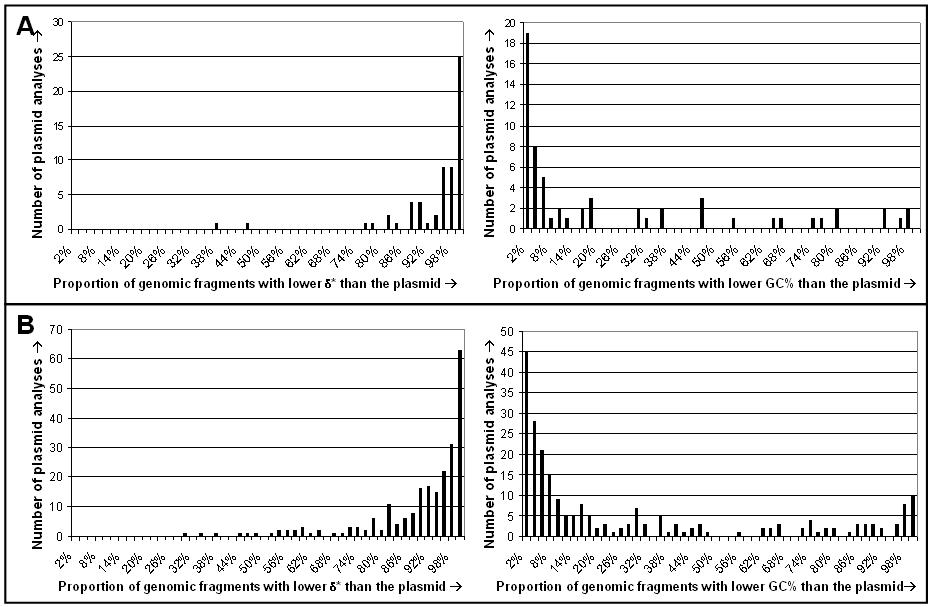
Distribution of the percentages of genomic fragments with a lower δ* or lower GC content than that of the plasmid. A) 61 plasmids compared to the genome sequence of the same strain. B) 230 plasmids compared with a single corresponding representative genome sequence.

Analyses of the δ* values between 61 plasmids and their corresponding host strains (comprising 30 prokaryotic species, Supplementary table S1 [see additional file 1]) show that in most instances the δ* between plasmid and the chromosome is higher than that of the bulk of the genomic fragments (Fig. 2A). Additionally, most of the plasmids have a lower GC content than the bulk of the chromosomal fragments of their respective hosts. Together these results indicate that the majority of plasmids have a DNA composition dissimilar to that of their corresponding host chromosome.

### Genome signature comparisons between plasmids and genomes of their host and relatives there off

For 21 prokaryotic species, of which plasmids are available in the plasmid genome database, different strains of the same species have been sequenced. The genome sequences of the strains belonging to the same species are compared to each other and the absolute δ* between these related chromosomes are depicted in table 1. In most cases, δ* values between the chromosome sequences of related strains within species are low (δ*<10), except for *Buchnera aphidicola *and *Pseudomonas syringae *(δ*>10). δ* values between 104 plasmids and chromosome sequences of the same (applicable) host species are comparable (supplementary table S2 [see additional file 1]), again except for *B. aphidicola *and *P. syringae *plasmids. This legitimizes the comparison of the nucleotide composition of plasmids, of which the host genome has not been sequenced, with that of a genome sequence of a representative strain.

### Genome signature comparisons between plasmids and genomes of a representative host

Finally, we compared the genomic dissimilarity between 230 plasmids from the Plasmid Genome Database and a single applicable representative chromosome each. In the case that multiple representative host chromosome sequences are available, a conservative choice was made (i.e. a representative host with the lowest δ* between the plasmid and genome sequence). For this analysis we excluded the different *B. aphidicola *and *P. syringae *plasmids, as no representative genome sequence can be selected due to high δ* values between chromosome sequences of members of the same species. Similar to the previous analysis, the genome signature of the majority of the plasmids exceeds that of the preponderance of the genomic fragments of each representative host chromosome, and has a lower GC content than the bulk of the chromosomal fragments of each representative host (Fig. 2B, supplementary table S3 [see additional file 1]). Also, we observe an increase in the number of plasmids with a very high GC content.

### Correlation between nucleotide composition discordance with host genomes and plasmid's size and mobility

**Figure 3:**
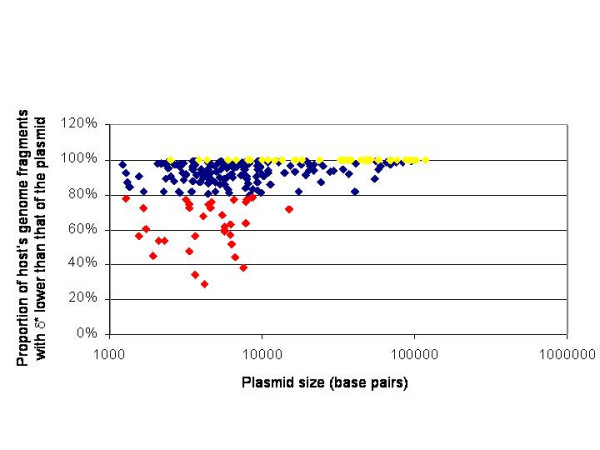
**Compositional discordance between plasmids and their host's according to the plasmid size. **The proportion of genomic fragments of the representative host chromosome with a δ* value lower than that of the plasmid is plotted as a function of plasmid size. Note the logarithmic scale on the X axis. Thirty-five plasmids have a δ* value lower than that of 80% (values range from 29% to 80%) of the identical sized fragments of their host's genome (red symbols), while 42 plasmids have a δ* value higher than all identical sized fragments of their host's genome (yellow symbols).

Of 230 plasmids, 195 have a δ* value higher than the δ* value of 80% of identical (to the plasmid) sized fragments of their host genome (Fig. 3), again indicating discordance in composition between plasmids and their host's genome. Of 230 plasmids, only 35 (15%) have a δ* value lower than that of 80% (values range from 29% to 80%) of the identical sized fragments of their host's genome. There was no relation with species of the host. Of these 35 plasmids, 18 have a size between 1 kbp and 5 kbp, 16 had a size between 5 kbp and 10 kbp, while only one was larger than 10 kbp. Of these 35 plasmids, eight (23%) harboured genes encoding putative proteins involved in mobility, another three (9%) had genes encoding putative proteins involved in transposition and five (14%) contained information encoding putative proteins involved in integration [13]. In contrast, of 230 plasmids, 42 have a δ* value higher than all identical sized fragments of their host's genome, indicating a high discordance between the nucleotide composition of these plasmids and that of their host genomes. The size of only three of these 42 plasmids ranged between 1 kbp and 5 kbp and that of only four between 5 kbp and 10 kbp. The remaining 35 plasmids with a high compositional discordance with their host's genome were larger than 10 kbp. Again, relation with species of the host was not observed. However, of these 42 plasmids, 17 (40%) harboured genes encoding putative proteins involved in mobility or transfer, while another eight (19%) encoded genes encoding putative proteins involved in transposition and only five (12%) contained information encoding putative proteins involved in integration.

## Discussion

In general, we find high genomic dissimilarity scores between plasmid sequences and representative host chromosome sequences. In addition, the GC contents of the plasmids show a bias towards low (and to a lesser extent, high) GC percentage scores. This lower GC content in plasmids has previously been noted, and has been explained in terms of a higher energy cost and limited availability of G and C over A and T/U [14]. Although available genome sequences are biased as they originate predominantly from medically and industrially relevant strains, it is unlikely that these plasmids form a particular class. In addition, our results are in accordance with those obtained by Wong and co-workers [10]. They showed, for a limited number of plasmids, that chromosomes within a species share a more similar dinucleotide composition, or genome signature, than plasmids do with the host chromosome(s).

Previously, Campell and co-workers compared plasmids to a collection of large chromosomal fragments of the host and showed that the genome signatures between each plasmid and its natural host rank amongst the closest [15]. Their suggestion that similar genome signatures of plasmids and host chromosome is required for plasmid establishment is not supported by the present data [15]. We find that intragenomic compositional comparisons of plasmids with their host often show higher genomic dissimilarity values than the genomic dissimilarity between genomic fragments and their host chromosome. This difference in interpretation of plasmid δ* values may be results of the, to our opinion more robust, method to compare these values with that of their host chromosome. First a distribution of δ* values by comparing disjoint genomic fragments to the full genomic sequence is made, providing information about the average and variance of the δ* values that a single species can display in different regions of its genome. Fragments with extreme δ* values (thus in the right tail of the distribution, Fig. 1) may result from events such as horizontal transfer or are caused by other genomic aberrations (e.g. rRNA gene clusters) [8,11]. Thus, these extreme fragments deviate substantially from the average genome composition and are considered compositionally dissimilar from the average chromosome content. Consequently, although the δ* values of most plasmids may fall within the very close category defined by Campbell and co-workers, we consider them as dissimilar, since they behave like the extreme fragments in the distribution plot. In addition, by comparing each plasmid with its host genome fragmented into pieces with the same size of the plasmid, the effect of the sensitivity of δ* of small DNA fragments to small changes in word is circumvented.

The genome signature of DNA is thought to have evolved due to selection exerted by its host's replication, recombination and repair machineries, resulting in comparable genome signatures between members of the same species, but different genome signatures between members of different species [6]. Plasmids seem to be less subjected to these selective pressures, although they are allegedly confined to a limited number of hosts due to the presence of partitioning genes and their dependence on the host replication machinery.

The observed genomic dissimilarity between the three different *B. aphidicola *genome sequences supports a role for replication, recombination and repair proteins in determining the genome signature. As the genome signature represents evolutionary relatedness between species similarly as other more classical parameters, such as 16S RNA similarity [16], intraspecific high genomic dissimilarity scores indicates rapid genome evolution or long-term host co-speciation (as has been described earlier [17]). The loss of genes involved in replication, recombination and the repair machinery in *Buchnera *genomes [18] might be responsible for the divergence of their genome signatures. These intracellular endosymbionts might then form an excellent example to investigate the origin of the genome signature. Interestingly, we find a *Buchnera *plasmid (plasmid pBBp1, NC_004555) which shows a high genomic dissimilarity with the genome sequence from the same strain from which the plasmid was isolated (i.e. *B. aphidicola *(*Baizongia pistaciae*)), and a lower genomic dissimilarity with both other *Buchnera *genome sequences. This supports a history of mobility for this plasmid, in which it was recently acquired from a different *Buchnera *strain, similar to previous observations by Van Ham and co-workers [19]. Interestingly, high genomic dissimilarity between members of the same genus (the Mollicutes) has been observed previously [20,21], which also concerns bacteria with an intracellular life-style.

We suggest three possible explanations for the reduced sensitivity of plasmids to the selective pressures generating their host's genome signature. First, the observed high genome signature dissimilarity may actually prevent the integration of plasmids into the host chromosome. Thus, what is observed for non-integrating plasmids in nature may be a biased pool of compositionally dissimilar DNA, as similar plasmids could potentially integrate into their host's chromosome more readily. Secondly, horizontally mobile plasmids may occasionally be exposed to the extracellular environment, where the atypical dinucleotide composition may favour resistance to degradation of the plasmid. Such a mechanism might drive the genome signature of plasmids towards comparable values, but the large variety in GC content among plasmids suggests otherwise. However, we cannot exclude that different environments select for different genome signatures. Thirdly, horizontal transmission of plasmids may be far more important than currently thought. This latter point is supported by the conclusion in a recent review by Sorensen and co-workers, that the overall extent of the HGT of plasmids in the environment examined might have been underestimated [22]. In addition, plasmid transfer between genera, phyla and even different domains has been described [22]. Plasmid transfer between unrelated species may be rare, but followed by a more rapid distribution among related species, would result in compositional discordance between many plasmids and their host. Our data, showing that a large proportion of the plasmids with high nucleotide discordance with their host's genome harbour genes encoding proteins involved in mobility or plasmid transfer, fits with this notion.

In addition, the plasmids showing relatively low nucleotide discordance with their host's genome are smaller than those showing high nucleotide discordance with their host's genome (Fig. 3). This could be indicative for a larger sensitivity of δ* of small DNA fragments to small changes in word than larger plasmids. However, 50% of the plasmids with a relatively low compositional discordance with their host's genome are larger than 5 kbp. Moreover, as aforementioned, the δ* value of each plasmid is compared with a distribution of δ* values of disjoint genomic fragments compared to the full genomic sequence, which provides information about the average and variance of the δ* values that in different regions of the host's genome. On the other hand, the copy number of small plasmids is in general higher than that of large plasmids. This would implicate faster replication of these smaller plasmids, hence faster amelioration rates.

We suggest that plasmids with high genomic dissimilarity scores are relatively recently acquired by the host, while the minority of plasmids with a genome signature similar to that of the host genome share a longer history with that host (i.e. a vertical association). The latter, strictly vertically transmitted, plasmids may therefore show a less atypical dinucleotide composition as a result from co-evolution with the host, but also selection due to extracellular conditions would be absent.

## Conclusion

The high genome signature divergence between plasmids and their hosts indicates that plasmids are excluded from the selective pressures that generate the genome signature, hence form a separate DNA flux within the global microbial metagenome. This suggests a more indiscriminate lifestyle for plasmids than previously anticipated.

## Methods

The approach is based on the dinucleotide relative abundance values or genome signature (ρ* _XY_). Karlin and Burge previously stated that each genome has its own genome signature, which is conserved between related species [5]. In brief, the dinucleotide relative abundance values ρ_XY _* are defined as the frequency of the dinucleotide XY divided by the product of the background frequencies of the individual nucleotides in the combined sense and reverse complement sequence (ρ* _XY _= f_XY_/(f_X _* f_Y_)). δ* is the average absolute dinucleotide relative abundance difference given by δ* (f, g) = 1/16 * ∑ | ρ_XY _*(f) - ρ_XY _*(g)|, where ρ_XY _*(f) denotes the abundance values calculated for input sequence f and ρ_XY _*(g) the abundance values calculated for genome sequence g. This calculation can be performed online at δρ-web [9] and also presents the amount of genomic fragments with a lower δ* or GC% [8]. All complete genome and plasmid sequences are retrieved from the NCBI [13] website as of 1 June 2005. To avoid statistically irrelevant computations, the minimum length of a plasmid sequence should be 1000 bp, allowing adequate dinucleotide counts per sequence. The maximum length of a plasmid sequence should not exceed 2% of that the host genome sequence, as longer sequences may not allow a genomic frequency distribution with ample genomic fragments [8,9]. Therefore plasmids smaller than 1000 bp and those larger than 2% of their host's genome were excluded.

## Authors' contributions

MvP, AB and AvdE devised the experimental setup and wrote the manuscript, and AL and AvK supplied the bioinformatical data acquisition.

## Supplementary Material

Additional File 1 Word document containing supplementary tables 1–3. Click here for file

## Figures and Tables

**Table 1:** Intraspecies genome signature comparisons. When more than 2 genome sequence are available for the plasmids from the database, the lowest and highest δ* is depicted (δ* _min _and δ* _max _respectively).

#	Species	# of available genome sequences*	δ*min (x1000)	δ*max (x1000)
1	*Bacillus anthracis*	3	0.012006	0.06936
2	*Bacillus cereus*	3	1.578633	4.59874
3	*Bacillus licheniformis*	2		0.05647
4	*Bacteroides fragilis*	2		1.859383
5	*Buchnera aphidicola*	3	16.95546	59.25254
6	*Campylobacter jejuni*	2		3.636911
7	*Escherichia coli*	4	0.596158	7.086455
8	*Helicobacter pylori*	2		7.324047
9	*Neisseria meningitidis*	2		3.94554
10	*Pseudomonas syringae*	2		16.17344
11	*Salmonella enterica*	2		1.560395
12	*Shigella flexneri*	2		0.365415
13	*Staphylococcus aureus*	6	0.761036	1.613145
14	*Staphylococcus epidermidis*	2		2.965408
15	*Streptococcus agalactiae*	2		3.441733
16	*Streptococcus pneumoniae*	2		2.326183
17	*Streptococcus pyogenes*	5	0.448649	2.864896
18	*Streptococcus thermophilus*	2		0.872791
19	*Thermus thermophilus*	2		1.734364
20	*Xylella fastidiosa*	2		6.746043
21	*Yersinia pestis*	3	0.335064	2.809856

*) At the time of analysis, June 2005
