# Supplementary material for: Compositional discordance between prokaryotic plasmids and host chromosomes
Source: BMC Genomics. 2006 Feb 15;7:26. doi: 10.1186/1471-2164-7-26 (PMC1382213; doi:10.1186/1471-2164-7-26)
Supplement: Additional File 1 — Word document containing supplementary tables 1–3. [file 1471-2164-7-26-S1.doc]

Supplementary table S1. Dinucleotide composition and GC percentage analyses of plasmid sequences with the genome sequences of the same strain. Depicted are the different plasmids (accession numbers), their length, their corresponding genome sequences (and accession number and size), the GC percentage of the plasmid, the plot position of the GC percentage analyses (ppGC), the genomic dissimilarity (δ*x1000) and the plot position of the analyses (ppδ*).

|  | **Plasmid accession number** | **Plasmid Name** | **Plasmid size (bp)** | **Genome accession number** | **Genome size (bp)** | **Organism** | **GC%** | **ppGC** | **δ***  **(x1000)** | **ppδ*** |
| --- | --- | --- | --- | --- | --- | --- | --- | --- | --- | --- |
| 30 | NC_004721 | pBClin15 | 15274 | NC_004722 | 5411809 | Bacillus cereus ATCC 14579 | 38% | 92% | 70 | 96% |
| 61 | NC_006578 | pBT9727 | 77112 | NC_005957 | 5237682 | Bacillus thuringiensis serovar konkukian str. 97-27 | 33% | 6% | 58 | 100% |
| 52 | NC_006297 | pBFY46 | 33716 | NC_006347 | 5277274 | Bacteroides fragilis YCH46 | 34% | 1% | 69 | 100% |
| 27 | NC_004703 | p5482 | 33038 | NC_004663 | 6260361 | Bacteroides thetaiotaomicron VPI-5482 | 47% | 98% | 94 | 100% |
| 34 | NC_004943 | pBLO1 | 3626 | NC_004307 | 2256646 | Bifidobacterium longum NCC2705 | 65% | 97% | 109 | 97% |
| 1 | NC_000957 | lp5 | 5228 | NC_001318 | 910724 | Borrelia burgdorferi B31 | 24% | 1% | 97 | 98% |
| 3 | NC_001849 | lp17 | 16823 | NC_001318 | 910724 | Borrelia burgdorferi B31 | 23% | 2% | 69 | 100% |
| 4 | NC_001904 | cp9 | 9386 | NC_001318 | 910724 | Borrelia burgdorferi B31 | 24% | 1% | 76 | 99% |
| 22 | NC_004555 | pBBp1 | 2399 | NC_004545 | 615980 | Buchnera aphidicola str. Bp (Baizongia pistaciae) | 25% | 53% | 150 | 100% |
| 5 | NC_002182 | pMoPn | 7501 | NC_002620 | 1072950 | Chlamydia muridarum Nigg | 36% | 1% | 30 | 38% |
| 9 | NC_003042 | pCP13 | 54310 | NC_003366 | 3031430 | Clostridium perfringens str. 13 | 26% | 2% | 39 | 89% |
| 28 | NC_004704 | pQpH1 | 37393 | NC_002971 | 1995275 | Coxiella burnetii RSA 493 | 39% | 2% | 62 | 100% |
| 25 | NC_004670 | pTEF3 | 17963 | NC_004668 | 3218031 | Enterococcus faecalis V583 | 33% | 4% | 72 | 97% |
| 26 | NC_004671 | pTEF2 | 57660 | NC_004668 | 3218031 | Enterococcus faecalis V583 | 34% | 4% | 54 | 95% |
| 60 | NC_006509 | pHTA426 | 47890 | NC_006510 | 3544776 | Geobacillus kaustophilus HTA426 | 44% | 1% | 123 | 100% |
| 55 | NC_006375 | pWCFS101 | 1917 | NC_004567 | 3308274 | Lactobacillus plantarum WCFS1 | 39% | 8% | 54 | 45% |
| 56 | NC_006376 | pWCFS102 | 2365 | NC_004567 | 3308274 | Lactobacillus plantarum WCFS1 | 34% | 1% | 122 | 98% |
| 57 | NC_006377 | pWCFS103 | 36069 | NC_004567 | 3308274 | Lactobacillus plantarum WCFS1 | 41% | 5% | 70 | 100% |
| 58 | NC_006399 | pPB1 | 2899 | NC_004567 | 3308274 | Lactobacillus plantarum WCFS1 | 38% | 4% | 110 | 98% |
| 54 | NC_006366 | pLPL | 59832 | NC_006369 | 3345687 | Legionella pneumophila str. Lens | 38% | 45% | 27 | 96% |
| 2 | NC_001733 | small extrachromosomal element | 16550 | NC_000909 | 1664970 | Methanocaldococcus jannaschii DSM 2661 | 29% | 3% | 62 | 93% |
| 53 | NC_006363 | pNF2 | 87093 | NC_006361 | 6021225 | Nocardia farcinica IFM 10152 | 68% | 3% | 33 | 100% |
| 13 | NC_003241 | pCC7120zeta | 5584 | NC_003272 | 6413771 | Nostoc sp. PCC 7120 | 44% | 91% | 76 | 97% |
| 14 | NC_003267 | pCC7120gamma | 101965 | NC_003272 | 6413771 | Nostoc sp. PCC 7120 | 41% | 29% | 40 | 100% |
| 15 | NC_003270 | pCC7120epsilon | 40340 | NC_003272 | 6413771 | Nostoc sp. PCC 7120 | 41% | 30% | 22 | 82% |
| 16 | NC_003273 | pCC7120 delta | 55414 | NC_003272 | 6413771 | Nostoc sp. PCC 7120 | 42% | 64% | 33 | 99% |
| 23 | NC_004632 | pDC3000B | 67473 | NC_004578 | 6397126 | Pseudomonas syringae pv. tomato str. DC3000 | 56% | 9% | 31 | 90% |
| 24 | NC_004633 | pDC3000A | 73661 | NC_004578 | 6397126 | Pseudomonas syringae pv. tomato str. DC3000 | 55% | 3% | 33 | 91% |
| 44 | NC_005297 | pRPA | 8427 | NC_005296 | 5459213 | Rhodopseudomonas palustris CGA009 | 60% | 2% | 160 | 100% |
| 17 | NC_003277 | pSLT | 93939 | NC_003197 | 4857432 | Salmonella typhimurium LT2 | 53% | 80% | 95 | 100% |
| 49 | NC_005951 | pSAS | 20652 | NC_002953 | 2799802 | Staphylococcus aureus subsp. aureus MSSA476 | 28% | 1% | 80 | 98% |
| 8 | NC_002774 | VRSAp | 25107 | NC_002758 | 2878529 | Staphylococcus aureus subsp. aureus Mu50 | 29% | 1% | 69 | 96% |
| 42 | NC_005011 | pMW2 | 20654 | NC_003923 | 2820462 | Staphylococcus aureus subsp. aureus MW2 | 28% | 1% | 81 | 99% |
| 12 | NC_003140 | pN315 | 24653 | NC_002745 | 2814816 | Staphylococcus aureus subsp. aureus N315 | 29% | 1% | 79 | 97% |
| 36 | NC_005003 | pSE-12228-06 | 6585 | NC_004461 | 2499279 | Staphylococcus epidermidis ATCC 12228 | 32% | 36% | 90 | 96% |
| 37 | NC_005004 | pSE-12228-05 | 24365 | NC_004461 | 2499279 | Staphylococcus epidermidis ATCC 12228 | 29% | 4% | 45 | 88% |
| 38 | NC_005005 | pSE-12228-04 | 17261 | NC_004461 | 2499279 | Staphylococcus epidermidis ATCC 12228 | 28% | 2% | 44 | 88% |
| 39 | NC_005006 | pSE-12228-03 | 8007 | NC_004461 | 2499279 | Staphylococcus epidermidis ATCC 12228 | 36% | 96% | 121 | 99% |
| 40 | NC_005007 | pSE-12228-02 | 4679 | NC_004461 | 2499279 | Staphylococcus epidermidis ATCC 12228 | 32% | 45% | 58 | 76% |
| 41 | NC_005008 | pSE-12228-01 | 4439 | NC_004461 | 2499279 | Staphylococcus epidermidis ATCC 12228 | 30% | 15% | 63 | 81% |
| 29 | NC_004719 | SAP1 | 94287 | NC_003155 | 9025608 | Streptomyces avermitilis MA-4680 | 69% | 11% | 53 | 99% |
| 35 | NC_004967 | pCB2.4 | 2345 | NC_000911 | 3573470 | Synechocystis sp. PCC 6803 | 43% | 10% | 80 | 89% |
| 43 | NC_005231 | pSYSG | 44343 | NC_000911 | 3573470 | Synechocystis sp. PCC 6803 | 49% | 75% | 118 | 100% |
| 45 | NC_005792 | pTT8 | 9328 | NC_006461 | 1849742 | Thermus thermophilus HB8 | 69% | 32% | 65 | 87% |
| 59 | NC_006463 | pTT8 | 9322 | NC_006461 | 1849742 | Thermus thermophilus HB8 | 69% | 35% | 65 | 87% |
| 18 | NC_003425 | pWb1 | 5280 | NC_004344 | 697724 | Wigglesworthia glossinidia endosymbiont of Glossina brevipalpis | 22% | 46% | 89 | 90% |
| 19 | NC_003921 | pXAC33 | 33700 | NC_003919 | 5175554 | Xanthomonas axonopodis pv. citri str. 306 | 62% | 5% | 64 | 95% |
| 20 | NC_003922 | pXAC64 | 64920 | NC_003919 | 5175554 | Xanthomonas axonopodis pv. citri str. 306 | 61% | 5% | 63 | 99% |
| 6 | NC_002489 | pXF1.3 | 1286 | NC_002488 | 2679306 | Xylella fastidiosa 9a5c | 56% | 74% | 99 | 78% |
| 7 | NC_002490 | pXF51 | 51158 | NC_002488 | 2679306 | Xylella fastidiosa 9a5c | 50% | 17% | 80 | 100% |
| 21 | NC_004554 | pXFPD1.3 | 1346 | NC_004556 | 2519802 | Xylella fastidiosa Temecula1 | 54% | 66% | 103 | 84% |
| 46 | NC_005813 | pCD1 | 70159 | NC_005810 | 4595065 | Yersinia pestis biovar Medievalis str. 91001 | 45% | 3% | 45 | 100% |
| 47 | NC_005814 | pCRY | 21742 | NC_005810 | 4595065 | Yersinia pestis biovar Medievalis str. 91001 | 49% | 79% | 47 | 97% |
| 48 | NC_005816 | pPCP1 | 9609 | NC_005810 | 4595065 | Yersinia pestis biovar Medievalis str. 91001 | 45% | 17% | 68 | 96% |
| 10 | NC_003131 | pCD1 | 70305 | NC_003143 | 4653728 | Yersinia pestis CO92 | 45% | 2% | 44 | 100% |
| 11 | NC_003132 | pPCP1 | 9612 | NC_003143 | 4653728 | Yersinia pestis CO92 | 45% | 16% | 67 | 96% |
| 31 | NC_004836 | pCD1 | 70504 | NC_004088 | 4600755 | Yersinia pestis KIM | 45% | 2% | 44 | 100% |
| 32 | NC_004837 | pPCP1 | 9610 | NC_004088 | 4600755 | Yersinia pestis KIM | 45% | 17% | 67 | 96% |
| 33 | NC_004839 | pCD1 | 70559 | NC_004088 | 4600755 | Yersinia pestis KIM | 45% | 2% | 44 | 100% |
| 50 | NC_006153 | pYV | 68526 | NC_006155 | 4744671 | Yersinia pseudotuberculosis IP 32953 | 45% | 1% | 46 | 100% |
| 51 | NC_006154 | pYptb32953 | 27702 | NC_006155 | 4744671 | Yersinia pseudotuberculosis IP 32953 | 45% | 6% | 44 | 93% |

Supplementary table S2. Dinucleotide composition and GC percentage analyses of plasmid sequences with multiple genome

sequences of a representative species. Depicted are the numbers, the number of different plasmids, the plasmid accession

numbers, plasmid lengths, the corresponding genome sequence accession number, size and organism, the GC percentage of the

plasmid, the amount of genomic fragments with a lower GC% (ppGC), the genomic dissimilarity (δ*x1000) and the amount of

genomic fragments with a lower δ* (ppδ*).

|  | # | **Plasmid accession number** | **Plasmid Name** | **Plasmid size (bp)** | **Genome accession number** | **Genome size (bp)** | Organism | **GC%** | **ppGC** | **δ***  **(x1000)** | **ppδ*** |
| --- | --- | --- | --- | --- | --- | --- | --- | --- | --- | --- | --- |
| 100 | 27 | NC_002146 | pX02 | 96231 | NC_003997 | 5227293 | Bacillus anthracis str. Ames | 33% | 7% | 43 | 100% |
| 101 |  | NC_002146 | pX02 | 96231 | NC_007530 | 5227419 | Bacillus anthracis str. 'Ames Ancestor' | 33% | 7% | 43 | 100% |
| 102 |  | NC_002146 | pX02 | 96231 | NC_005945 | 5228663 | Bacillus anthracis str. Sterne | 33% | 7% | 42 | 100% |
| 192 | 51 | NC_004721 | pBClin15 | 15274 | NC_003909 | 5224283 | Bacillus cereus ATCC 10987 | 38% | 92% | 73 | 98% |
| 193 |  | NC_004721 | pBClin15 | 15274 | NC_004722 | 5411809 | Bacillus cereus ATCC 14579 | 38% | 92% | 70 | 96% |
| 194 |  | NC_004721 | pBClin15 | 15274 | NC_006274 | 5300915 | Bacillus cereus ZK | 38% | 93% | 73 | 98% |
| 283 | 82 | NC_005308 | pFL7 | 7853 | NC_006270 | 4222336 | Bacillus licheniformis ATCC 14580 | 44% | 12% | 66 | 91% |
| 286 |  | NC_005311 | pFL5 | 9150 | NC_006270 | 4222336 | Bacillus licheniformis ATCC 14580 | 41% | 5% | 63 | 90% |
| 284 |  | NC_005308 | pFL7 | 7853 | NC_006322 | 4222645 | Bacillus licheniformis DSM 13 | 44% | 12% | 66 | 91% |
| 285 | 83 | NC_005311 | pFL5 | 9150 | NC_006322 | 4222645 | Bacillus licheniformis DSM 13 | 41% | 5% | 63 | 90% |
| 263 | 77 | NC_005026 | pBI143 | 2747 | NC_003228 | 5205140 | Bacteroides fragilis NCTC 9343 | 41% | 30% | 100 | 98% |
| 264 |  | NC_005026 | pBI143 | 2747 | NC_006347 | 5277274 | Bacteroides fragilis YCH46 | 41% | 29% | 101 | 97% |
| 35 | 11 | NC_001910 | plasmid pLeu-Sg | 7967 | NC_002528 | 640681 | Buchnera aphidicola str. APS (Acyrthosiphon pisum) | 27% | 68% | 42 | 69% |
| 38 | 12 | NC_001911 | pLeu-Dn | 7768 | NC_002528 | 640681 | Buchnera aphidicola str. APS (Acyrthosiphon pisum) | 26% | 45% | 50 | 82% |
| 179 | 47 | NC_004555 | pBBp1 | 2399 | NC_002528 | 640681 | Buchnera aphidicola str. APS (Acyrthosiphon pisum) | 25% | 37% | 109 | 97% |
| 210 | 58 | NC_004843 | pBPS1 | 2308 | NC_002528 | 640681 | Buchnera aphidicola str. APS (Acyrthosiphon pisum) | 24% | 24% | 90 | 87% |
| 36 |  | NC_001910 | pLeu-Sg | 7967 | NC_004545 | 615980 | Buchnera aphidicola str. Bp (Baizongia pistaciae) | 27% | 81% | 60 | 95% |
| 39 |  | NC_001911 | pLeu-Dn | 7768 | NC_004545 | 615980 | Buchnera aphidicola str. Bp (Baizongia pistaciae) | 26% | 63% | 65 | 95% |
| 180 |  | NC_004555 | pBBp1 | 2399 | NC_004545 | 615980 | Buchnera aphidicola str. Bp (Baizongia pistaciae) | 25% | 53% | 150 | 100% |
| 211 |  | NC_004843 | pBPS1 | 2308 | NC_004545 | 615980 | Buchnera aphidicola str. Bp (Baizongia pistaciae) | 24% | 37% | 120 | 97% |
| 37 |  | NC_001910 | pLeu-Sg | 7967 | NC_004061 | 641454 | Buchnera aphidicola str. Sg (Schizaphis graminum) | 27% | 78% | 42 | 64% |
| 40 |  | NC_001911 | pLeu-Dn | 7768 | NC_004061 | 641454 | Buchnera aphidicola str. Sg (Schizaphis graminum) | 26% | 68% | 54 | 83% |
| 181 |  | NC_004555 | pBBp1 | 2399 | NC_004061 | 641454 | Buchnera aphidicola str. Sg (Schizaphis graminum) | 25% | 53% | 95 | 90% |
| 212 |  | NC_004843 | pBPS1 | 2308 | NC_004061 | 641454 | Buchnera aphidicola str. Sg (Schizaphis graminum) | 24% | 37% | 83 | 76% |
| 223 | 64 | NC_004997 | pCJ419 | 4013 | NC_003912 | 1777831 | Campylobacter jejuni RM1221 | 30% | 47% | 87 | 93% |
| 224 |  | NC_004997 | pCJ419 | 4013 | NC_002163 | 1641481 | Campylobacter jejuni subsp. jejuni NCTC 11168 | 30% | 45% | 88 | 93% |
| 7 | 4 | NC_001537 | pECO29 | 3895 | NC_004431 | 5231428 | Escherichia coli CFT073 | 46% | 11% | 78 | 87% |
| 69 | 19 | NC_002119 | CloDF13 | 9957 | NC_004431 | 5231428 | Escherichia coli CFT073 | 54% | 95% | 77 | 92% |
| 73 | 20 | NC_002127 | pOSAK1 | 3306 | NC_004431 | 5231428 | Escherichia coli CFT073 | 43% | 7% | 64 | 74% |
| 77 | 21 | NC_002128 | pO157 | 92721 | NC_004431 | 5231428 | Escherichia coli CFT073 | 48% | 4% | 64 | 100% |
| 89 | 24 | NC_002142 | pB171 | 68817 | NC_004431 | 5231428 | Escherichia coli CFT073 | 46% | 1% | 63 | 97% |
| 96 | 26 | NC_002145 | pKL1 | 1549 | NC_004431 | 5231428 | Escherichia coli CFT073 | 51% | 45% | 71 | 61% |
| 115 | 30 | NC_002482 | pGBG1 | 7620 | NC_004431 | 5231428 | Escherichia coli CFT073 | 52% | 74% | 80 | 92% |
| 119 | 31 | NC_002487 | pCol-let | 5847 | NC_004431 | 5231428 | Escherichia coli CFT073 | 47% | 13% | 77 | 90% |
| 131 | 34 | NC_002525 | R721 | 75582 | NC_004431 | 5231428 | Escherichia coli CFT073 | 43% | 1% | 57 | 99% |
| 167 | 43 | NC_003435 | pCA4 | 3078 | NC_004431 | 5231428 | Escherichia coli CFT073 | 50% | 37% | 87 | 89% |
| 173 | 45 | NC_004429 | pIS2 | 6349 | NC_004431 | 5231428 | Escherichia coli CFT073 | 47% | 14% | 41 | 55% |
| 243 | 72 | NC_005019 | pLG13 | 6293 | NC_004431 | 5231428 | Escherichia coli CFT073 | 44% | 4% | 97 | 96% |
| 279 | 81 | NC_005248 | pIGAL1 | 8145 | NC_004431 | 5231428 | Escherichia coli CFT073 | 51% | 41% | 64 | 87% |
| 293 |  | NC_005324 | p9123 | 6222 | NC_004431 | 5231428 | Escherichia coli CFT073 | 52% | 72% | 47 | 64% |
| 295 | 87 | NC_005327 | pC15-1a | 92353 | NC_004431 | 5231428 | Escherichia coli CFT073 | 53% | 98% | 50 | 100% |
| 315 |  | NC_005568 | pBHRK18 | 5721 | NC_004431 | 5231428 | Escherichia coli CFT073 | 57% | 98% | 47 | 64% |
| 319 |  | NC_005569 | pBHRK19 | 5721 | NC_004431 | 5231428 | Escherichia coli CFT073 | 57% | 98% | 47 | 62% |
| 344 | 100 | NC_005923 | pFL129 | 6464 | NC_004431 | 5231428 | Escherichia coli CFT073 | 52% | 74% | 73 | 90% |
| 354 | 102 | NC_005970 | pRK2 | 5360 | NC_004431 | 5231428 | Escherichia coli CFT073 | 46% | 11% | 102 | 97% |
| 8 |  | NC_001537 | pECO29 | 3895 | NC_000913 | 4639675 | Escherichia coli K12 | 46% | 9% | 81 | 94% |
| 70 |  | NC_002119 | CloDF13 | 9957 | NC_000913 | 4639675 | Escherichia coli K12 | 54% | 95% | 78 | 98% |
| 74 |  | NC_002127 | pOSAK1 | 3306 | NC_000913 | 4639675 | Escherichia coli K12 | 43% | 5% | 65 | 84% |
| 78 |  | NC_002128 | pO157 | 92721 | NC_000913 | 4639675 | Escherichia coli K12 | 48% | 2% | 72 | 100% |
| 90 |  | NC_002142 | pB171 | 68817 | NC_000913 | 4639675 | Escherichia coli K12 | 46% | 1% | 70 | 100% |
| 97 |  | NC_002145 | pKL1 | 1549 | NC_000913 | 4639675 | Escherichia coli K12 | 51% | 44% | 74 | 69% |
| 116 |  | NC_002482 | pGBG1 | 7620 | NC_000913 | 4639675 | Escherichia coli K12 | 52% | 70% | 80 | 97% |
| 120 |  | NC_002487 | pCol-let | 5847 | NC_000913 | 4639675 | Escherichia coli K12 | 47% | 10% | 82 | 97% |
| 132 |  | NC_002525 | R721 | 75582 | NC_000913 | 4639675 | Escherichia coli K12 | 43% | 2% | 64 | 100% |
| 168 |  | NC_003435 | pCA4 | 3078 | NC_000913 | 4639675 | Escherichia coli K12 | 50% | 35% | 94 | 96% |
| 174 |  | NC_004429 | pIS2 | 6349 | NC_000913 | 4639675 | Escherichia coli K12 | 47% | 13% | 48 | 77% |
| 244 |  | NC_005019 | pLG13 | 6293 | NC_000913 | 4639675 | Escherichia coli K12 | 44% | 3% | 102 | 99% |
| 280 |  | NC_005248 | pIGAL1 | 8145 | NC_000913 | 4639675 | Escherichia coli K12 | 51% | 38% | 68 | 95% |
| 291 | 86 | NC_005324 | p9123 | 6222 | NC_000913 | 4639675 | Escherichia coli K12 | 52% | 71% | 51 | 80% |
| 296 |  | NC_005327 | pC15-1a | 92353 | NC_000913 | 4639675 | Escherichia coli K12 | 53% | 100% | 58 | 100% |
| 316 |  | NC_005568 | pBHRK18 | 5721 | NC_000913 | 4639675 | Escherichia coli K12 | 57% | 99% | 50 | 78% |
| 320 |  | NC_005569 | pBHRK19 | 5721 | NC_000913 | 4639675 | Escherichia coli K12 | 57% | 99% | 50 | 77% |
| 347 |  | NC_005923 | pFL129 | 6464 | NC_000913 | 4639675 | Escherichia coli K12 | 52% | 70% | 74 | 96% |
| 357 |  | NC_005970 | pRK2 | 5360 | NC_000913 | 4639675 | Escherichia coli K12 | 46% | 8% | 104 | 99% |
| 9 |  | NC_001537 | pECO29 | 3895 | NC_002695 | 5498450 | Escherichia coli O157:H7 | 46% | 11% | 76 | 88% |
| 71 |  | NC_002119 | CloDF13 | 9957 | NC_002695 | 5498450 | Escherichia coli O157:H7 | 54% | 93% | 74 | 95% |
| 75 |  | NC_002127 | pOSAK1 | 3306 | NC_002695 | 5498450 | Escherichia coli O157:H7 | 43% | 7% | 63 | 72% |
| 79 |  | NC_002128 | pO157 | 92721 | NC_002695 | 5498450 | Escherichia coli O157:H7 | 48% | 5% | 65 | 100% |
| 91 |  | NC_002142 | pB171 | 68817 | NC_002695 | 5498450 | Escherichia coli O157:H7 | 46% | 1% | 63 | 100% |
| 98 |  | NC_002145 | pKL1 | 1549 | NC_002695 | 5498450 | Escherichia coli O157:H7 | 51% | 44% | 70 | 56% |
| 117 |  | NC_002482 | pGBG1 | 7620 | NC_002695 | 5498450 | Escherichia coli O157:H7 | 52% | 71% | 79 | 94% |
| 121 |  | NC_002487 | pCol-let | 5847 | NC_002695 | 5498450 | Escherichia coli O157:H7 | 47% | 13% | 76 | 91% |
| 133 |  | NC_002525 | R721 | 75582 | NC_002695 | 5498450 | Escherichia coli O157:H7 | 43% | 1% | 58 | 100% |
| 169 |  | NC_003435 | pCA4 | 3078 | NC_002695 | 5498450 | Escherichia coli O157:H7 | 50% | 36% | 88 | 91% |
| 175 |  | NC_004429 | pIS2 | 6349 | NC_002695 | 5498450 | Escherichia coli O157:H7 | 47% | 16% | 42 | 55% |
| 245 |  | NC_005019 | pLG13 | 6293 | NC_002695 | 5498450 | Escherichia coli O157:H7 | 44% | 6% | 96 | 97% |
| 281 |  | NC_005248 | pIGAL1 | 8145 | NC_002695 | 5498450 | Escherichia coli O157:H7 | 51% | 42% | 63 | 86% |
| 292 |  | NC_005324 | p9123 | 6222 | NC_002695 | 5498450 | Escherichia coli O157:H7 | 52% | 70% | 47 | 64% |
| 297 |  | NC_005327 | pC15-1a | 92353 | NC_002695 | 5498450 | Escherichia coli O157:H7 | 53% | 93% | 51 | 100% |
| 314 |  | NC_005568 | pBHRK18 | 5721 | NC_002695 | 5498450 | Escherichia coli O157:H7 | 57% | 97% | 47 | 61% |
| 318 |  | NC_005569 | pBHRK19 | 5721 | NC_002695 | 5498450 | Escherichia coli O157:H7 | 57% | 97% | 46 | 60% |
| 346 |  | NC_005923 | pFL129 | 6464 | NC_002695 | 5498450 | Escherichia coli O157:H7 | 52% | 70% | 72 | 90% |
| 356 |  | NC_005970 | pRK2 | 5360 | NC_002695 | 5498450 | Escherichia coli O157:H7 | 46% | 11% | 100 | 97% |
| 10 |  | NC_001537 | pECO29 | 3895 | NC_002655 | 5528445 | Escherichia coli O157:H7 EDL933 | 46% | 13% | 76 | 87% |
| 72 |  | NC_002119 | CloDF13 | 9957 | NC_002655 | 5528445 | Escherichia coli O157:H7 EDL933 | 54% | 93% | 74 | 93% |
| 76 |  | NC_002127 | pOSAK1 | 3306 | NC_002655 | 5528445 | Escherichia coli O157:H7 EDL933 | 43% | 7% | 64 | 72% |
| 80 |  | NC_002128 | pO157 | 92721 | NC_002655 | 5528445 | Escherichia coli O157:H7 EDL933 | 48% | 8% | 64 | 100% |
| 92 |  | NC_002142 | pB171 | 68817 | NC_002655 | 5528445 | Escherichia coli O157:H7 EDL933 | 46% | 1% | 63 | 100% |
| 99 |  | NC_002145 | pKL1 | 1549 | NC_002655 | 5528445 | Escherichia coli O157:H7 EDL933 | 51% | 45% | 70 | 57% |
| 118 |  | NC_002482 | pGBG1 | 7620 | NC_002655 | 5528445 | Escherichia coli O157:H7 EDL933 | 52% | 71% | 79 | 94% |
| 122 |  | NC_002487 | pCol-let | 5847 | NC_002655 | 5528445 | Escherichia coli O157:H7 EDL933 | 47% | 14% | 75 | 91% |
| 134 |  | NC_002525 | R721 | 75582 | NC_002655 | 5528445 | Escherichia coli O157:H7 EDL933 | 43% | 1% | 57 | 100% |
| 170 |  | NC_003435 | pCA4 | 3078 | NC_002655 | 5528445 | Escherichia coli O157:H7 EDL933 | 50% | 37% | 88 | 90% |
| 176 |  | NC_004429 | pIS2 | 6349 | NC_002655 | 5528445 | Escherichia coli O157:H7 EDL933 | 47% | 16% | 41 | 52% |
| 246 |  | NC_005019 | pLG13 | 6293 | NC_002655 | 5528445 | Escherichia coli O157:H7 EDL933 | 44% | 6% | 96 | 97% |
| 282 |  | NC_005248 | pIGAL1 | 8145 | NC_002655 | 5528445 | Escherichia coli O157:H7 EDL933 | 51% | 43% | 62 | 87% |
| 294 |  | NC_005324 | p9123 | 6222 | NC_002655 | 5528445 | Escherichia coli O157:H7 EDL933 | 52% | 71% | 47 | 63% |
| 298 |  | NC_005327 | pC15-1a | 92353 | NC_002655 | 5528445 | Escherichia coli O157:H7 EDL933 | 53% | 97% | 51 | 100% |
| 313 | 91 | NC_005568 | pBHRK18 | 5721 | NC_002655 | 5528445 | Escherichia coli O157:H7 EDL933 | 57% | 98% | 47 | 60% |
| 317 | 92 | NC_005569 | pBHRK19 | 5721 | NC_002655 | 5528445 | Escherichia coli O157:H7 EDL933 | 57% | 98% | 46 | 59% |
| 345 |  | NC_005923 | pFL129 | 6464 | NC_002655 | 5528445 | Escherichia coli O157:H7 EDL933 | 52% | 72% | 72 | 90% |
| 355 |  | NC_005970 | pRK2 | 5360 | NC_002655 | 5528445 | Escherichia coli O157:H7 EDL933 | 46% | 12% | 100 | 97% |
| 5 | 3 | NC_001476 | pHPM186 | 12887 | NC_000915 | 1667867 | Helicobacter pylori 26695 | 36% | 6% | 104 | 99% |
| 17 | 6 | NC_001756 | pHPM180 | 3506 | NC_000915 | 1667867 | Helicobacter pylori 26695 | 37% | 19% | 104 | 95% |
| 33 | 10 | NC_001843 | pHP489 | 1222 | NC_000915 | 1667867 | Helicobacter pylori 26695 | 33% | 6% | 158 | 97% |
| 67 | 18 | NC_002110 | pHPO100 | 3520 | NC_000915 | 1667867 | Helicobacter pylori 26695 | 36% | 11% | 105 | 96% |
| 199 | 54 | NC_004767 | pHP51 | 3955 | NC_000915 | 1667867 | Helicobacter pylori 26695 | 35% | 7% | 90 | 95% |
| 213 | 59 | NC_004845 | pHPM8 | 7817 | NC_000915 | 1667867 | Helicobacter pylori 26695 | 33% | 3% | 120 | 99% |
| 215 | 60 | NC_004949 | pHel5 | 18291 | NC_000915 | 1667867 | Helicobacter pylori 26695 | 34% | 2% | 136 | 100% |
| 217 | 61 | NC_004950 | pHel4 | 10970 | NC_000915 | 1667867 | Helicobacter pylori 26695 | 34% | 5% | 138 | 100% |
| 342 | 99 | NC_005917 | pAL202 | 12120 | NC_000915 | 1667867 | Helicobacter pylori 26695 | 34% | 4% | 147 | 100% |
| 343 |  | NC_005917 | pAL202 | 12120 | NC_000921 | 1643831 | Helicobacter pylori J99 | 34% | 1% | 150 | 100% |
| 6 |  | NC_001476 | pHPM186 | 12887 | NC_000921 | 1643831 | Helicobacter pylori, strain J99 complete genome | 36% | 3% | 107 | 98% |
| 18 |  | NC_001756 | pHPM180 | 3506 | NC_000921 | 1643831 | Helicobacter pylori, strain J99 complete genome | 37% | 15% | 106 | 95% |
| 34 |  | NC_001843 | pHP489 | 1222 | NC_000921 | 1643831 | Helicobacter pylori, strain J99 complete genome | 33% | 4% | 158 | 97% |
| 68 |  | NC_002110 | pHPO100 | 3520 | NC_000921 | 1643831 | Helicobacter pylori, strain J99 complete genome | 36% | 8% | 108 | 95% |
| 200 |  | NC_004767 | pHP51 | 3955 | NC_000921 | 1643831 | Helicobacter pylori, strain J99 complete genome | 35% | 5% | 94 | 95% |
| 214 |  | NC_004845 | pHPM8 | 7817 | NC_000921 | 1643831 | Helicobacter pylori, strain J99 complete genome | 33% | 1% | 123 | 99% |
| 216 |  | NC_004949 | pHel5 | 18291 | NC_000921 | 1643831 | Helicobacter pylori, strain J99 complete genome | 34% | 1% | 140 | 100% |
| 218 |  | NC_004950 | pHel4 | 10970 | NC_000921 | 1643831 | Helicobacter pylori, strain J99 complete genome | 34% | 1% | 140 | 100% |
| 197 | 53 | NC_004758 | pJS-B | 7245 | NC_003112 | 2272351 | Neisseria meningitidis MC58 | 42% | 3% | 86 | 91% |
| 198 |  | NC_004758 | pJS-B | 7245 | NC_003116 | 2184406 | Neisseria meningitidis Z2491 | 42% | 2% | 89 | 96% |
| 188 | 49 | NC_004632 | pDC3000B | 67473 | NC_007005 | 6093698 | Pseudomonas syringae pv. syringae B728a | 56% | 6% | 38 | 96% |
| 190 | 50 | NC_004633 | pDC3000A | 73661 | NC_007005 | 6093698 | Pseudomonas syringae pv. syringae B728a | 55% | 1% | 40 | 94% |
| 189 |  | NC_004632 | pDC3000B | 67473 | NC_004578 | 6397126 | Pseudomonas syringae pv. tomato str. DC3000 | 56% | 9% | 31 | 90% |
| 191 |  | NC_004633 | pDC3000A | 73661 | NC_004578 | 6397126 | Pseudomonas syringae pv. tomato str. DC3000 | 55% | 3% | 33 | 91% |
| 135 | 35 | NC_002638 | pKDSC50 | 49503 | NC_006905 | 4755700 | Salmonella enterica subsp. enterica serovar Choleraesuis str. SC-B67 | 52% | 41% | 97 | 100% |
| 332 | 97 | NC_005862 | cryptic plasmid | 6066 | NC_006905 | 4755700 | Salmonella enterica subsp. enterica serovar Choleraesuis str. SC-B67 | 54% | 63% | 62 | 86% |
| 136 |  | NC_002638 | pKDSC50 | 49503 | NC_006511 | 4585229 | Salmonella enterica subsp. enterica serovar Paratypi A str. ATCC 9150 | 52% | 38% | 99 | 100% |
| 333 |  | NC_005862 | cryptic plasmid | 6066 | NC_006511 | 4585229 | Salmonella enterica subsp. enterica serovar Paratypi A str. ATCC 9150 | 54% | 62% | 63 | 87% |
| 137 |  | NC_002638 | pKDSC50 | 49503 | NC_003198 | 4809037 | Salmonella enterica subsp. enterica serovar Typhi str. CT18 | 52% | 40% | 95 | 100% |
| 334 |  | NC_005862 | cryptic plasmid | 6066 | NC_003198 | 4809037 | Salmonella enterica subsp. enterica serovar Typhi str. CT18 | 54% | 64% | 59 | 82% |
| 138 |  | NC_002638 | pKDSC50 | 49503 | NC_004631 | 4791961 | Salmonella enterica subsp. enterica serovar Typhi Ty2 | 52% | 43% | 95 | 100% |
| 335 |  | NC_005862 | cryptic plasmid | 6066 | NC_004631 | 4791961 | Salmonella enterica subsp. enterica serovar Typhi Ty2 | 54% | 62% | 60 | 81% |
| 139 | 36 | NC_002773 | p2457TS2 | 3179 | NC_004741 | 4599354 | Shigella flexneri 2a str. 2457T | 45% | 5% | 62 | 77% |
| 140 |  | NC_002773 | p2457TS2 | 3179 | NC_004337 | 4607203 | Shigella flexneri 2a str. 301 | 45% | 5% | 62 | 77% |
| 11 | 5 | NC_001565 | pUB110 | 2266 | NC_002951 | 2809422 | Staphylococcus aureus subsp. aureus COL | 36% | 87% | 90 | 83% |
| 19 | 7 | NC_001763 | J3358 | 6024 | NC_002951 | 2809422 | Staphylococcus aureus subsp. aureus COL | 31% | 20% | 69 | 86% |
| 25 | 8 | NC_001767 | pKH6 | 4439 | NC_002951 | 2809422 | Staphylococcus aureus subsp. aureus COL | 30% | 9% | 81 | 89% |
| 41 | 13 | NC_001994 | pSK3 | 1658 | NC_002951 | 2809422 | Staphylococcus aureus subsp. aureus COL | 34% | 69% | 121 | 92% |
| 47 | 14 | NC_001995 | pSK6 | 1551 | NC_002951 | 2809422 | Staphylococcus aureus subsp. aureus COL | 34% | 61% | 118 | 91% |
| 53 | 15 | NC_002013 | pC194 | 2910 | NC_002951 | 2809422 | Staphylococcus aureus subsp. aureus COL | 29% | 8% | 125 | 96% |
| 61 | 17 | NC_002096 | pKH7 | 4118 | NC_002951 | 2809422 | Staphylococcus aureus subsp. aureus COL | 29% | 4% | 62 | 74% |
| 81 | 22 | NC_002129 | pC221 | 4557 | NC_002951 | 2809422 | Staphylococcus aureus subsp. aureus COL | 32% | 28% | 63 | 75% |
| 103 | 28 | NC_002156 | J3356::POX7;1 | 8007 | NC_002951 | 2809422 | Staphylococcus aureus subsp. aureus COL | 39% | 98% | 72 | 91% |
| 109 | 29 | NC_002157 | J3356::pOX7;3 | 8007 | NC_002951 | 2809422 | Staphylococcus aureus subsp. aureus COL | 38% | 98% | 71 | 90% |
| 125 | 33 | NC_002517 | p21 | 20719 | NC_002951 | 2809422 | Staphylococcus aureus subsp. aureus COL | 29% | 1% | 82 | 97% |
| 141 | 37 | NC_002774 | VRSAp | 25107 | NC_002951 | 2809422 | Staphylococcus aureus subsp. aureus COL | 29% | 1% | 70 | 99% |
| 155 | 41 | NC_003140 | pN315 | 24653 | NC_002951 | 2809422 | Staphylococcus aureus subsp. aureus COL | 29% | 1% | 80 | 100% |
| 161 | 42 | NC_003265 | pETB | 38211 | NC_002951 | 2809422 | Staphylococcus aureus subsp. aureus COL | 28% | 1% | 92 | 100% |
| 182 | 48 | NC_004562 | pNVH01 | 2650 | NC_002951 | 2809422 | Staphylococcus aureus subsp. aureus COL | 30% | 16% | 96 | 90% |
| 237 | 71 | NC_005011 | pMW2 | 20654 | NC_002951 | 2809422 | Staphylococcus aureus subsp. aureus COL | 28% | 1% | 81 | 99% |
| 247 | 73 | NC_005020 | pKH3 | 2979 | NC_002951 | 2809422 | Staphylococcus aureus subsp. aureus COL | 29% | 6% | 104 | 93% |
| 257 | 76 | NC_005024 | pSK41 | 46445 | NC_002951 | 2809422 | Staphylococcus aureus subsp. aureus COL | 30% | 2% | 75 | 100% |
| 269 |  | NC_005127 | pUB101 | 21845 | NC_002951 | 2809422 | Staphylococcus aureus subsp. aureus COL | 29% | 1% | 70 | 95% |
| 274 |  | NC_005243 | pC223 | 4608 | NC_002951 | 2809422 | Staphylococcus aureus subsp. aureus COL | 31% | 25% | 83 | 89% |
| 299 | 88 | NC_005564 | pS194 | 4397 | NC_002951 | 2809422 | Staphylococcus aureus subsp. aureus COL | 32% | 26% | 72 | 83% |
| 305 | 89 | NC_005565 | pSN2 | 1288 | NC_002951 | 2809422 | Staphylococcus aureus subsp. aureus COL | 36% | 90% | 135 | 93% |
| 340 |  | NC_005908 | pE194 | 3728 | NC_002951 | 2809422 | Staphylococcus aureus subsp. aureus COL | 32% | 31% | 92 | 91% |
| 348 | 101 | NC_005951 | pSAS | 20652 | NC_002951 | 2809422 | Staphylococcus aureus subsp. aureus COL | 28% | 1% | 81 | 99% |
| 12 |  | NC_001565 | pUB110 | 2266 | NC_002952 | 2902619 | Staphylococcus aureus subsp. aureus MRSA252 | 36% | 88% | 89 | 83% |
| 20 |  | NC_001763 | J3358 | 6024 | NC_002952 | 2902619 | Staphylococcus aureus subsp. aureus MRSA252 | 31% | 19% | 68 | 83% |
| 26 |  | NC_001767 | pKH6 | 4439 | NC_002952 | 2902619 | Staphylococcus aureus subsp. aureus MRSA252 | 30% | 9% | 80 | 86% |
| 42 |  | NC_001994 | pSK3 | 1658 | NC_002952 | 2902619 | Staphylococcus aureus subsp. aureus MRSA252 | 34% | 70% | 120 | 92% |
| 48 |  | NC_001995 | pSK6 | 1551 | NC_002952 | 2902619 | Staphylococcus aureus subsp. aureus MRSA252 | 34% | 62% | 117 | 91% |
| 54 |  | NC_002013 | pC194 | 2910 | NC_002952 | 2902619 | Staphylococcus aureus subsp. aureus MRSA252 | 29% | 8% | 124 | 96% |
| 62 |  | NC_002096 | pKH7 | 4118 | NC_002952 | 2902619 | Staphylococcus aureus subsp. aureus MRSA252 | 29% | 4% | 61 | 69% |
| 82 |  | NC_002129 | pC221 | 4557 | NC_002952 | 2902619 | Staphylococcus aureus subsp. aureus MRSA252 | 32% | 29% | 61 | 73% |
| 104 |  | NC_002156 | J3356::POX7;1 | 8007 | NC_002952 | 2902619 | Staphylococcus aureus subsp. aureus MRSA252 | 39% | 99% | 70 | 89% |
| 110 |  | NC_002157 | J3356::pOX7;3 | 8007 | NC_002952 | 2902619 | Staphylococcus aureus subsp. aureus MRSA252 | 38% | 99% | 70 | 89% |
| 126 |  | NC_002517 | p21 | 20719 | NC_002952 | 2902619 | Staphylococcus aureus subsp. aureus MRSA252 | 29% | 1% | 80 | 98% |
| 142 |  | NC_002774 | VRSAp | 25107 | NC_002952 | 2902619 | Staphylococcus aureus subsp. aureus MRSA252 | 29% | 1% | 69 | 99% |
| 156 |  | NC_003140 | pN315 | 24653 | NC_002952 | 2902619 | Staphylococcus aureus subsp. aureus MRSA252 | 29% | 1% | 78 | 98% |
| 162 |  | NC_003265 | pETB | 38211 | NC_002952 | 2902619 | Staphylococcus aureus subsp. aureus MRSA252 | 28% | 1% | 91 | 100% |
| 183 |  | NC_004562 | pNVH01 | 2650 | NC_002952 | 2902619 | Staphylococcus aureus subsp. aureus MRSA252 | 30% | 15% | 95 | 89% |
| 238 |  | NC_005011 | pMW2 | 20654 | NC_002952 | 2902619 | Staphylococcus aureus subsp. aureus MRSA252 | 28% | 1% | 80 | 99% |
| 248 |  | NC_005020 | pKH3 | 2979 | NC_002952 | 2902619 | Staphylococcus aureus subsp. aureus MRSA252 | 29% | 6% | 103 | 92% |
| 258 |  | NC_005024 | pSK41 | 46445 | NC_002952 | 2902619 | Staphylococcus aureus subsp. aureus MRSA252 | 30% | 2% | 74 | 100% |
| 272 |  | NC_005127 | pUB101 | 21845 | NC_002952 | 2902619 | Staphylococcus aureus subsp. aureus MRSA252 | 29% | 1% | 69 | 98% |
| 275 |  | NC_005243 | pC223 | 4608 | NC_002952 | 2902619 | Staphylococcus aureus subsp. aureus MRSA252 | 31% | 25% | 82 | 89% |
| 300 |  | NC_005564 | pS194 | 4397 | NC_002952 | 2902619 | Staphylococcus aureus subsp. aureus MRSA252 | 32% | 27% | 70 | 80% |
| 306 |  | NC_005565 | pSN2 | 1288 | NC_002952 | 2902619 | Staphylococcus aureus subsp. aureus MRSA252 | 36% | 89% | 134 | 93% |
| 339 |  | NC_005908 | pE194 | 3728 | NC_002952 | 2902619 | Staphylococcus aureus subsp. aureus MRSA252 | 32% | 31% | 90 | 91% |
| 349 |  | NC_005951 | pSAS | 20652 | NC_002952 | 2902619 | Staphylococcus aureus subsp. aureus MRSA252 | 28% | 1% | 79 | 99% |
| 13 |  | NC_001565 | pUB110 | 2266 | NC_002953 | 2799802 | Staphylococcus aureus subsp. aureus MSSA476 | 36% | 88% | 90 | 84% |
| 21 |  | NC_001763 | J3358 | 6024 | NC_002953 | 2799802 | Staphylococcus aureus subsp. aureus MSSA476 | 31% | 19% | 69 | 86% |
| 27 |  | NC_001767 | pKH6 | 4439 | NC_002953 | 2799802 | Staphylococcus aureus subsp. aureus MSSA476 | 30% | 9% | 80 | 88% |
| 43 |  | NC_001994 | pSK3 | 1658 | NC_002953 | 2799802 | Staphylococcus aureus subsp. aureus MSSA476 | 34% | 70% | 121 | 92% |
| 49 |  | NC_001995 | pSK6 | 1551 | NC_002953 | 2799802 | Staphylococcus aureus subsp. aureus MSSA476 | 34% | 62% | 118 | 90% |
| 55 |  | NC_002013 | pC194 | 2910 | NC_002953 | 2799802 | Staphylococcus aureus subsp. aureus MSSA476 | 29% | 7% | 125 | 96% |
| 63 |  | NC_002096 | pKH7 | 4118 | NC_002953 | 2799802 | Staphylococcus aureus subsp. aureus MSSA476 | 29% | 4% | 62 | 71% |
| 83 |  | NC_002129 | pC221 | 4557 | NC_002953 | 2799802 | Staphylococcus aureus subsp. aureus MSSA476 | 32% | 28% | 62 | 74% |
| 105 |  | NC_002156 | J3356::POX7;1 | 8007 | NC_002953 | 2799802 | Staphylococcus aureus subsp. aureus MSSA476 | 39% | 98% | 71 | 90% |
| 111 |  | NC_002157 | J3356::pOX7;3 | 8007 | NC_002953 | 2799802 | Staphylococcus aureus subsp. aureus MSSA476 | 38% | 98% | 71 | 90% |
| 127 |  | NC_002517 | p21 | 20719 | NC_002953 | 2799802 | Staphylococcus aureus subsp. aureus MSSA476 | 29% | 1% | 81 | 99% |
| 143 |  | NC_002774 | VRSAp | 25107 | NC_002953 | 2799802 | Staphylococcus aureus subsp. aureus MSSA476 | 29% | 1% | 70 | 96% |
| 157 |  | NC_003140 | pN315 | 24653 | NC_002953 | 2799802 | Staphylococcus aureus subsp. aureus MSSA476 | 29% | 1% | 79 | 99% |
| 163 |  | NC_003265 | pETB | 38211 | NC_002953 | 2799802 | Staphylococcus aureus subsp. aureus MSSA476 | 28% | 1% | 92 | 100% |
| 184 |  | NC_004562 | pNVH01 | 2650 | NC_002953 | 2799802 | Staphylococcus aureus subsp. aureus MSSA476 | 30% | 15% | 95 | 89% |
| 239 |  | NC_005011 | pMW2 | 20654 | NC_002953 | 2799802 | Staphylococcus aureus subsp. aureus MSSA476 | 28% | 1% | 81 | 99% |
| 249 |  | NC_005020 | pKH3 | 2979 | NC_002953 | 2799802 | Staphylococcus aureus subsp. aureus MSSA476 | 29% | 6% | 103 | 92% |
| 259 |  | NC_005024 | pSK41 | 46445 | NC_002953 | 2799802 | Staphylococcus aureus subsp. aureus MSSA476 | 30% | 2% | 75 | 100% |
| 268 |  | NC_005127 | pUB101 | 21845 | NC_002953 | 2799802 | Staphylococcus aureus subsp. aureus MSSA476 | 29% | 1% | 70 | 95% |
| 276 |  | NC_005243 | pC223 | 4608 | NC_002953 | 2799802 | Staphylococcus aureus subsp. aureus MSSA476 | 31% | 25% | 83 | 90% |
| 301 |  | NC_005564 | pS194 | 4397 | NC_002953 | 2799802 | Staphylococcus aureus subsp. aureus MSSA476 | 32% | 26% | 71 | 82% |
| 307 |  | NC_005565 | pSN2 | 1288 | NC_002953 | 2799802 | Staphylococcus aureus subsp. aureus MSSA476 | 36% | 89% | 134 | 93% |
| 341 |  | NC_005908 | pE194 | 3728 | NC_002953 | 2799802 | Staphylococcus aureus subsp. aureus MSSA476 | 32% | 30% | 91 | 92% |
| 350 |  | NC_005951 | pSAS | 20652 | NC_002953 | 2799802 | Staphylococcus aureus subsp. aureus MSSA476 | 28% | 1% | 80 | 98% |
| 14 |  | NC_001565 | pUB110 | 2266 | NC_002758 | 2878529 | Staphylococcus aureus subsp. aureus Mu50 | 36% | 88% | 89 | 81% |
| 22 |  | NC_001763 | J3358 | 6024 | NC_002758 | 2878529 | Staphylococcus aureus subsp. aureus Mu50 | 31% | 17% | 69 | 85% |
| 28 |  | NC_001767 | pKH6 | 4439 | NC_002758 | 2878529 | Staphylococcus aureus subsp. aureus Mu50 | 30% | 8% | 80 | 87% |
| 44 |  | NC_001994 | pSK3 | 1658 | NC_002758 | 2878529 | Staphylococcus aureus subsp. aureus Mu50 | 34% | 69% | 120 | 92% |
| 50 |  | NC_001995 | pSK6 | 1551 | NC_002758 | 2878529 | Staphylococcus aureus subsp. aureus Mu50 | 34% | 61% | 118 | 91% |
| 56 |  | NC_002013 | pC194 | 2910 | NC_002758 | 2878529 | Staphylococcus aureus subsp. aureus Mu50 | 29% | 7% | 124 | 96% |
| 64 |  | NC_002096 | pKH7 | 4118 | NC_002758 | 2878529 | Staphylococcus aureus subsp. aureus Mu50 | 29% | 3% | 61 | 68% |
| 84 |  | NC_002129 | pC221 | 4557 | NC_002758 | 2878529 | Staphylococcus aureus subsp. aureus Mu50 | 32% | 25% | 62 | 75% |
| 106 |  | NC_002156 | J3356::POX7;1 | 8007 | NC_002758 | 2878529 | Staphylococcus aureus subsp. aureus Mu50 | 39% | 98% | 70 | 90% |
| 112 |  | NC_002157 | J3356::pOX7;3 | 8007 | NC_002758 | 2878529 | Staphylococcus aureus subsp. aureus Mu50 | 38% | 98% | 70 | 89% |
| 128 |  | NC_002517 | p21 | 20719 | NC_002758 | 2878529 | Staphylococcus aureus subsp. aureus Mu50 | 29% | 1% | 80 | 98% |
| 144 |  | NC_002774 | VRSAp | 25107 | NC_002758 | 2878529 | Staphylococcus aureus subsp. aureus Mu50 | 29% | 1% | 69 | 96% |
| 158 |  | NC_003140 | pN315 | 24653 | NC_002758 | 2878529 | Staphylococcus aureus subsp. aureus Mu50 | 29% | 1% | 78 | 97% |
| 164 |  | NC_003265 | pETB | 38211 | NC_002758 | 2878529 | Staphylococcus aureus subsp. aureus Mu50 | 28% | 1% | 91 | 100% |
| 185 |  | NC_004562 | pNVH01 | 2650 | NC_002758 | 2878529 | Staphylococcus aureus subsp. aureus Mu50 | 30% | 14% | 95 | 88% |
| 240 |  | NC_005011 | pMW2 | 20654 | NC_002758 | 2878529 | Staphylococcus aureus subsp. aureus Mu50 | 28% | 1% | 80 | 96% |
| 250 |  | NC_005020 | pKH3 | 2979 | NC_002758 | 2878529 | Staphylococcus aureus subsp. aureus Mu50 | 29% | 5% | 104 | 93% |
| 260 |  | NC_005024 | pSK41 | 46445 | NC_002758 | 2878529 | Staphylococcus aureus subsp. aureus Mu50 | 30% | 2% | 74 | 100% |
| 267 | 79 | NC_005127 | pUB101 | 21845 | NC_002758 | 2878529 | Staphylococcus aureus subsp. aureus Mu50 | 29% | 1% | 69 | 94% |
| 273 | 80 | NC_005243 | pC223 | 4608 | NC_002758 | 2878529 | Staphylococcus aureus subsp. aureus Mu50 | 31% | 26% | 83 | 88% |
| 302 |  | NC_005564 | pS194 | 4397 | NC_002758 | 2878529 | Staphylococcus aureus subsp. aureus Mu50 | 32% | 26% | 71 | 82% |
| 308 |  | NC_005565 | pSN2 | 1288 | NC_002758 | 2878529 | Staphylococcus aureus subsp. aureus Mu50 | 36% | 89% | 134 | 93% |
| 336 | 98 | NC_005908 | pE194 | 3728 | NC_002758 | 2878529 | Staphylococcus aureus subsp. aureus Mu50 | 32% | 30% | 91 | 90% |
| 351 |  | NC_005951 | pSAS | 20652 | NC_002758 | 2878529 | Staphylococcus aureus subsp. aureus Mu50 | 28% | 1% | 80 | 97% |
| 15 |  | NC_001565 | pUB110 | 2266 | NC_003923 | 2820462 | Staphylococcus aureus subsp. aureus MW2 | 36% | 88% | 90 | 83% |
| 23 |  | NC_001763 | J3358 | 6024 | NC_003923 | 2820462 | Staphylococcus aureus subsp. aureus MW2 | 31% | 21% | 68 | 85% |
| 29 |  | NC_001767 | pKH6 | 4439 | NC_003923 | 2820462 | Staphylococcus aureus subsp. aureus MW2 | 30% | 11% | 80 | 88% |
| 45 |  | NC_001994 | pSK3 | 1658 | NC_003923 | 2820462 | Staphylococcus aureus subsp. aureus MW2 | 34% | 68% | 121 | 92% |
| 51 |  | NC_001995 | pSK6 | 1551 | NC_003923 | 2820462 | Staphylococcus aureus subsp. aureus MW2 | 34% | 63% | 118 | 90% |
| 57 |  | NC_002013 | pC194 | 2910 | NC_003923 | 2820462 | Staphylococcus aureus subsp. aureus MW2 | 29% | 8% | 125 | 97% |
| 65 |  | NC_002096 | pKH7 | 4118 | NC_003923 | 2820462 | Staphylococcus aureus subsp. aureus MW2 | 29% | 4% | 62 | 70% |
| 85 |  | NC_002129 | pC221 | 4557 | NC_003923 | 2820462 | Staphylococcus aureus subsp. aureus MW2 | 32% | 28% | 62 | 76% |
| 107 |  | NC_002156 | J3356::POX7;1 | 8007 | NC_003923 | 2820462 | Staphylococcus aureus subsp. aureus MW2 | 39% | 99% | 71 | 90% |
| 113 |  | NC_002157 | J3356::pOX7;3 | 8007 | NC_003923 | 2820462 | Staphylococcus aureus subsp. aureus MW2 | 38% | 99% | 71 | 90% |
| 129 |  | NC_002517 | p21 | 20719 | NC_003923 | 2820462 | Staphylococcus aureus subsp. aureus MW2 | 29% | 1% | 81 | 99% |
| 145 |  | NC_002774 | VRSAp | 25107 | NC_003923 | 2820462 | Staphylococcus aureus subsp. aureus MW2 | 29% | 1% | 70 | 96% |
| 159 |  | NC_003140 | pN315 | 24653 | NC_003923 | 2820462 | Staphylococcus aureus subsp. aureus MW2 | 29% | 1% | 79 | 99% |
| 165 |  | NC_003265 | pETB | 38211 | NC_003923 | 2820462 | Staphylococcus aureus subsp. aureus MW2 | 28% | 1% | 92 | 100% |
| 186 |  | NC_004562 | pNVH01 | 2650 | NC_003923 | 2820462 | Staphylococcus aureus subsp. aureus MW2 | 30% | 15% | 96 | 88% |
| 241 |  | NC_005011 | pMW2 | 20654 | NC_003923 | 2820462 | Staphylococcus aureus subsp. aureus MW2 | 28% | 1% | 81 | 99% |
| 251 |  | NC_005020 | pKH3 | 2979 | NC_003923 | 2820462 | Staphylococcus aureus subsp. aureus MW2 | 29% | 6% | 103 | 92% |
| 261 |  | NC_005024 | pSK41 | 46445 | NC_003923 | 2820462 | Staphylococcus aureus subsp. aureus MW2 | 30% | 2% | 75 | 100% |
| 270 |  | NC_005127 | pUB101 | 21845 | NC_003923 | 2820462 | Staphylococcus aureus subsp. aureus MW2 | 29% | 1% | 70 | 95% |
| 277 |  | NC_005243 | pC223 | 4608 | NC_003923 | 2820462 | Staphylococcus aureus subsp. aureus MW2 | 31% | 23% | 83 | 90% |
| 303 |  | NC_005564 | pS194 | 4397 | NC_003923 | 2820462 | Staphylococcus aureus subsp. aureus MW2 | 32% | 26% | 71 | 82% |
| 309 |  | NC_005565 | pSN2 | 1288 | NC_003923 | 2820462 | Staphylococcus aureus subsp. aureus MW2 | 36% | 90% | 134 | 92% |
| 337 |  | NC_005908 | pE194 | 3728 | NC_003923 | 2820462 | Staphylococcus aureus subsp. aureus MW2 | 32% | 30% | 91 | 91% |
| 352 |  | NC_005951 | pSAS | 20652 | NC_003923 | 2820462 | Staphylococcus aureus subsp. aureus MW2 | 28% | 1% | 81 | 99% |
| 16 |  | NC_001565 | pUB110 | 2266 | NC_002745 | 2814816 | Staphylococcus aureus subsp. aureus N315 | 36% | 88% | 89 | 83% |
| 24 |  | NC_001763 | J3358 | 6024 | NC_002745 | 2814816 | Staphylococcus aureus subsp. aureus N315 | 31% | 18% | 69 | 85% |
| 30 |  | NC_001767 | pKH6 | 4439 | NC_002745 | 2814816 | Staphylococcus aureus subsp. aureus N315 | 30% | 9% | 80 | 87% |
| 46 |  | NC_001994 | pSK3 | 1658 | NC_002745 | 2814816 | Staphylococcus aureus subsp. aureus N315 | 34% | 69% | 120 | 91% |
| 52 |  | NC_001995 | pSK6 | 1551 | NC_002745 | 2814816 | Staphylococcus aureus subsp. aureus N315 | 34% | 62% | 118 | 90% |
| 58 |  | NC_002013 | pC194 | 2910 | NC_002745 | 2814816 | Staphylococcus aureus subsp. aureus N315 | 29% | 7% | 125 | 96% |
| 66 |  | NC_002096 | pKH7 | 4118 | NC_002745 | 2814816 | Staphylococcus aureus subsp. aureus N315 | 29% | 4% | 61 | 70% |
| 86 |  | NC_002129 | pC221 | 4557 | NC_002745 | 2814816 | Staphylococcus aureus subsp. aureus N315 | 32% | 27% | 62 | 74% |
| 108 |  | NC_002156 | J3356::POX7;1 | 8007 | NC_002745 | 2814816 | Staphylococcus aureus subsp. aureus N315 | 39% | 98% | 71 | 90% |
| 114 |  | NC_002157 | J3356::pOX7;3 | 8007 | NC_002745 | 2814816 | Staphylococcus aureus subsp. aureus N315 | 38% | 98% | 71 | 90% |
| 130 |  | NC_002517 | p21 | 20719 | NC_002745 | 2814816 | Staphylococcus aureus subsp. aureus N315 | 29% | 1% | 81 | 99% |
| 146 |  | NC_002774 | VRSAp | 25107 | NC_002745 | 2814816 | Staphylococcus aureus subsp. aureus N315 | 29% | 1% | 69 | 98% |
| 160 |  | NC_003140 | pN315 | 24653 | NC_002745 | 2814816 | Staphylococcus aureus subsp. aureus N315 | 29% | 1% | 79 | 97% |
| 166 |  | NC_003265 | pETB | 38211 | NC_002745 | 2814816 | Staphylococcus aureus subsp. aureus N315 | 28% | 1% | 91 | 100% |
| 187 |  | NC_004562 | pNVH01 | 2650 | NC_002745 | 2814816 | Staphylococcus aureus subsp. aureus N315 | 30% | 15% | 96 | 89% |
| 242 |  | NC_005011 | pMW2 | 20654 | NC_002745 | 2814816 | Staphylococcus aureus subsp. aureus N315 | 28% | 1% | 80 | 99% |
| 252 |  | NC_005020 | pKH3 | 2979 | NC_002745 | 2814816 | Staphylococcus aureus subsp. aureus N315 | 29% | 5% | 104 | 92% |
| 262 |  | NC_005024 | pSK41 | 46445 | NC_002745 | 2814816 | Staphylococcus aureus subsp. aureus N315 | 30% | 2% | 75 | 100% |
| 271 |  | NC_005127 | pUB101 | 21845 | NC_002745 | 2814816 | Staphylococcus aureus subsp. aureus N315 | 29% | 1% | 69 | 98% |
| 278 |  | NC_005243 | pC223 | 4608 | NC_002745 | 2814816 | Staphylococcus aureus subsp. aureus N315 | 31% | 22% | 83 | 90% |
| 304 |  | NC_005564 | pS194 | 4397 | NC_002745 | 2814816 | Staphylococcus aureus subsp. aureus N315 | 32% | 26% | 71 | 82% |
| 310 |  | NC_005565 | pSN2 | 1288 | NC_002745 | 2814816 | Staphylococcus aureus subsp. aureus N315 | 36% | 89% | 134 | 93% |
| 338 |  | NC_005908 | pE194 | 3728 | NC_002745 | 2814816 | Staphylococcus aureus subsp. aureus N315 | 32% | 31% | 91 | 91% |
| 353 |  | NC_005951 | pSAS | 20652 | NC_002745 | 2814816 | Staphylococcus aureus subsp. aureus N315 | 28% | 1% | 80 | 99% |
| 171 | 44 | NC_003969 | pSepCH | 2391 | NC_004461 | 2499279 | Staphylococcus epidermidis ATCC 12228 | 29% | 12% | 146 | 98% |
| 225 | 65 | NC_005003 | pSE-12228-06 | 6585 | NC_004461 | 2499279 | Staphylococcus epidermidis ATCC 12228 | 32% | 36% | 90 | 96% |
| 227 | 66 | NC_005004 | pSE-12228-05 | 24365 | NC_004461 | 2499279 | Staphylococcus epidermidis ATCC 12228 | 29% | 4% | 45 | 88% |
| 229 | 67 | NC_005005 | pSE-12228-04 | 17261 | NC_004461 | 2499279 | Staphylococcus epidermidis ATCC 12228 | 28% | 2% | 44 | 88% |
| 231 | 68 | NC_005006 | pSE-12228-03 | 8007 | NC_004461 | 2499279 | Staphylococcus epidermidis ATCC 12228 | 36% | 96% | 121 | 99% |
| 233 | 69 | NC_005007 | pSE-12228-02 | 4679 | NC_004461 | 2499279 | Staphylococcus epidermidis ATCC 12228 | 32% | 45% | 58 | 76% |
| 235 | 70 | NC_005008 | pSE-12228-01 | 4439 | NC_004461 | 2499279 | Staphylococcus epidermidis ATCC 12228 | 30% | 15% | 63 | 81% |
| 311 | 90 | NC_005566 | pSK639 | 8013 | NC_004461 | 2499279 | Staphylococcus epidermidis ATCC 12228 | 34% | 87% | 48 | 77% |
| 172 |  | NC_003969 | pSepCH | 2391 | NC_002976 | 2616530 | Staphylococcus epidermidis RP62A | 29% | 10% | 147 | 98% |
| 226 |  | NC_005003 | pSE-12228-06 | 6585 | NC_002976 | 2616530 | Staphylococcus epidermidis RP62A | 32% | 36% | 91 | 95% |
| 228 |  | NC_005004 | pSE-12228-05 | 24365 | NC_002976 | 2616530 | Staphylococcus epidermidis RP62A | 29% | 4% | 44 | 87% |
| 230 |  | NC_005005 | pSE-12228-04 | 17261 | NC_002976 | 2616530 | Staphylococcus epidermidis RP62A | 28% | 1% | 43 | 82% |
| 232 |  | NC_005006 | pSE-12228-03 | 8007 | NC_002976 | 2616530 | Staphylococcus epidermidis RP62A | 36% | 96% | 122 | 99% |
| 234 |  | NC_005007 | pSE-12228-02 | 4679 | NC_002976 | 2616530 | Staphylococcus epidermidis RP62A | 32% | 44% | 60 | 75% |
| 236 |  | NC_005008 | pSE-12228-01 | 4439 | NC_002976 | 2616530 | Staphylococcus epidermidis RP62A | 30% | 14% | 61 | 74% |
| 312 |  | NC_005566 | pSK639 | 8013 | NC_002976 | 2616530 | Staphylococcus epidermidis RP62A | 34% | 89% | 50 | 78% |
| 31 | 9 | NC_001797 | pGB354 | 6437 | NC_004116 | 2160267 | Streptococcus agalactiae 2603V/R | 33% | 7% | 97 | 99% |
| 87 | 23 | NC_002136 | pGB3631 | 5842 | NC_004116 | 2160267 | Streptococcus agalactiae 2603V/R | 34% | 13% | 95 | 98% |
| 32 |  | NC_001797 | pGB354 | 6437 | NC_004368 | 2211485 | Streptococcus agalactiae NEM316 | 33% | 7% | 97 | 98% |
| 88 |  | NC_002136 | pGB3631 | 5842 | NC_004368 | 2211485 | Streptococcus agalactiae NEM316 | 34% | 13% | 97 | 97% |
| 253 | 74 | NC_005021 | pSMB1 | 3162 | NC_003098 | 2038615 | Streptococcus pneumoniae R6 complete genome | 32% | 5% | 86 | 92% |
| 255 | 75 | NC_005022 | pDP1 | 3161 | NC_003098 | 2038615 | Streptococcus pneumoniae R6 complete genome | 32% | 5% | 85 | 92% |
| 254 |  | NC_005021 | pSMB1 | 3162 | NC_003028 | 2160837 | Streptococcus pneumoniae TIGR4 | 32% | 4% | 87 | 92% |
| 256 |  | NC_005022 | pDP1 | 3161 | NC_003028 | 2160837 | Streptococcus pneumoniae TIGR4 | 32% | 4% | 87 | 91% |
| 362 |  | NC_006130 | pDN571 | 3351 | NC_002737 | 1852441 | Streptococcus pyogenes M1 GAS | 34% | 6% | 53 | 55% |
| 361 |  | NC_006130 | pDN571 | 3351 | NC_006086 | 1899877 | Streptococcus pyogenes MGAS10394 | 34% | 6% | 52 | 51% |
| 360 |  | NC_006130 | pDN571 | 3351 | NC_004070 | 1900521 | Streptococcus pyogenes MGAS315 | 34% | 5% | 51 | 48% |
| 358 | 103 | NC_006130 | pDN571 | 3351 | NC_004606 | 1894275 | Streptococcus pyogenes SSI-1 | 34% | 6% | 51 | 47% |
| 359 |  | NC_006130 | pDN571 | 3351 | NC_003485 | 1895017 | Streptococcus pyogenes strain MGAS8232 | 34% | 6% | 51 | 47% |
| 1 | 1 | NC_000937 | pER35 | 9531 | NC_006449 | 1796226 | Streptococcus thermophilus CNRZ1066 | 37% | 10% | 89 | 99% |
| 3 | 2 | NC_000938 | pER36 | 3498 | NC_006449 | 1796226 | Streptococcus thermophilus CNRZ1066 | 34% | 5% | 77 | 88% |
| 147 | 38 | NC_002776 | pER13 | 4139 | NC_006449 | 1796226 | Streptococcus thermophilus CNRZ1066 | 38% | 36% | 74 | 90% |
| 195 | 52 | NC_004747 | pND103 | 3531 | NC_006449 | 1796226 | Streptococcus thermophilus CNRZ1066 | 32% | 4% | 123 | 99% |
| 219 | 62 | NC_004958 | pSMQ172 | 4230 | NC_006449 | 1796226 | Streptococcus thermophilus CNRZ1066 | 38% | 28% | 79 | 92% |
| 221 | 63 | NC_004968 | pER371 | 2672 | NC_006449 | 1796226 | Streptococcus thermophilus CNRZ1066 | 38% | 32% | 115 | 97% |
| 265 | 78 | NC_005098 | pt38 | 2911 | NC_006449 | 1796226 | Streptococcus thermophilus CNRZ1066 | 32% | 4% | 72 | 81% |
| 287 | 84 | NC_005322 | pSMQ308 | 8144 | NC_006449 | 1796226 | Streptococcus thermophilus CNRZ1066 | 38% | 24% | 173 | 100% |
| 289 | 85 | NC_005323 | pSMQ173b | 4449 | NC_006449 | 1796226 | Streptococcus thermophilus CNRZ1066 | 37% | 15% | 85 | 94% |
| 2 |  | NC_000937 | pER35 | 9531 | NC_006448 | 1796846 | Streptococcus thermophilus LMG 18311 | 37% | 10% | 90 | 99% |
| 4 |  | NC_000938 | pER36 | 3498 | NC_006448 | 1796846 | Streptococcus thermophilus LMG 18311 | 34% | 6% | 77 | 88% |
| 148 |  | NC_002776 | pER13 | 4139 | NC_006448 | 1796846 | Streptococcus thermophilus LMG 18311 | 38% | 35% | 75 | 90% |
| 196 |  | NC_004747 | pND103 | 3531 | NC_006448 | 1796846 | Streptococcus thermophilus LMG 18311 | 32% | 3% | 123 | 99% |
| 220 |  | NC_004958 | pSMQ172 | 4230 | NC_006448 | 1796846 | Streptococcus thermophilus LMG 18311 | 38% | 29% | 80 | 91% |
| 222 |  | NC_004968 | pER371 | 2672 | NC_006448 | 1796846 | Streptococcus thermophilus LMG 18311 | 38% | 33% | 116 | 97% |
| 266 |  | NC_005098 | pt38 | 2911 | NC_006448 | 1796846 | Streptococcus thermophilus LMG 18311 | 32% | 3% | 72 | 81% |
| 288 |  | NC_005322 | pSMQ308 | 8144 | NC_006448 | 1796846 | Streptococcus thermophilus LMG 18311 | 38% | 24% | 173 | 100% |
| 290 |  | NC_005323 | pSMQ173b | 4449 | NC_006448 | 1796846 | Streptococcus thermophilus LMG 18311 | 37% | 15% | 85 | 95% |
| 321 | 93 | NC_005792 | pTT8 | 9328 | NC_005835 | 1894877 | Thermus thermophilus HB27 | 69% | 30% | 65 | 90% |
| 363 | 104 | NC_006463 | pTT8 | 9322 | NC_005835 | 1894877 | Thermus thermophilus HB27 | 69% | 33% | 65 | 89% |
| 322 |  | NC_005792 | pTT8 | 9328 | NC_006461 | 1849742 | Thermus thermophilus HB8 | 69% | 32% | 65 | 87% |
| 364 |  | NC_006463 | pTT8 | 9322 | NC_006461 | 1849742 | Thermus thermophilus HB8 | 69% | 35% | 65 | 87% |
| 59 | 16 | NC_002092 | pXF868 | 1296 | NC_002488 | 2679306 | Xylella fastidiosa 9a5c | 55% | 69% | 111 | 87% |
| 123 | 32 | NC_002489 | pXF1.3 | 1286 | NC_002488 | 2679306 | Xylella fastidiosa 9a5c | 56% | 74% | 99 | 78% |
| 177 | 46 | NC_004554 | pXFPD1.3 | 1346 | NC_002488 | 2679306 | Xylella fastidiosa 9a5c | 54% | 59% | 105 | 84% |
| 60 |  | NC_002092 | pXF868 | 1296 | NC_004556 | 2519802 | Xylella fastidiosa Temecula1 | 55% | 74% | 109 | 87% |
| 124 |  | NC_002489 | pXF1.3 | 1286 | NC_004556 | 2519802 | Xylella fastidiosa Temecula1 | 56% | 79% | 97 | 79% |
| 178 |  | NC_004554 | pXFPD1.3 | 1346 | NC_004556 | 2519802 | Xylella fastidiosa Temecula1 | 54% | 66% | 103 | 84% |
| 93 | 25 | NC_002144 | pYC | 5919 | NC_005810 | 4595065 | Yersinia pestis biovar Medievalis str. 91001 | 40% | 3% | 82 | 97% |
| 149 | 39 | NC_003131 | pCD1 | 70305 | NC_005810 | 4595065 | Yersinia pestis biovar Medievalis str. 91001 | 45% | 5% | 45 | 100% |
| 152 | 40 | NC_003132 | pPCP1 | 9612 | NC_005810 | 4595065 | Yersinia pestis biovar Medievalis str. 91001 | 45% | 18% | 67 | 96% |
| 201 | 55 | NC_004836 | pCD1 | 70504 | NC_005810 | 4595065 | Yersinia pestis biovar Medievalis str. 91001 | 45% | 5% | 45 | 100% |
| 204 | 56 | NC_004837 | pPCP1 | 9610 | NC_005810 | 4595065 | Yersinia pestis biovar Medievalis str. 91001 | 45% | 18% | 68 | 96% |
| 207 | 57 | NC_004839 | pCD1 | 70559 | NC_005810 | 4595065 | Yersinia pestis biovar Medievalis str. 91001 | 45% | 5% | 45 | 100% |
| 323 | 94 | NC_005813 | pCD1 | 70159 | NC_005810 | 4595065 | Yersinia pestis biovar Medievalis str. 91001 | 45% | 3% | 45 | 100% |
| 326 | 95 | NC_005814 | pCRY | 21742 | NC_005810 | 4595065 | Yersinia pestis biovar Medievalis str. 91001 | 49% | 79% | 47 | 97% |
| 329 | 96 | NC_005816 | pPCP1 | 9609 | NC_005810 | 4595065 | Yersinia pestis biovar Medievalis str. 91001 | 45% | 17% | 68 | 96% |
| 94 |  | NC_002144 | pYC | 5919 | NC_003143 | 4653728 | Yersinia pestis CO92 | 40% | 3% | 81 | 96% |
| 150 |  | NC_003131 | pCD1 | 70305 | NC_003143 | 4653728 | Yersinia pestis CO92 | 45% | 2% | 44 | 100% |
| 153 |  | NC_003132 | pPCP1 | 9612 | NC_003143 | 4653728 | Yersinia pestis CO92 | 45% | 16% | 67 | 96% |
| 202 |  | NC_004836 | pCD1 | 70504 | NC_003143 | 4653728 | Yersinia pestis CO92 | 45% | 2% | 44 | 100% |
| 205 |  | NC_004837 | pPCP1 | 9610 | NC_003143 | 4653728 | Yersinia pestis CO92 | 45% | 16% | 67 | 96% |
| 208 |  | NC_004839 | pCD1 | 70559 | NC_003143 | 4653728 | Yersinia pestis CO92 | 45% | 2% | 44 | 100% |
| 324 |  | NC_005813 | pCD1 | 70159 | NC_003143 | 4653728 | Yersinia pestis CO92 | 45% | 2% | 44 | 100% |
| 327 |  | NC_005814 | pCRY | 21742 | NC_003143 | 4653728 | Yersinia pestis CO92 | 49% | 78% | 47 | 96% |
| 330 |  | NC_005816 | pPCP1 | 9609 | NC_003143 | 4653728 | Yersinia pestis CO92 | 45% | 17% | 67 | 96% |
| 95 |  | NC_002144 | pYC | 5919 | NC_004088 | 4600755 | Yersinia pestis KIM | 40% | 2% | 81 | 96% |
| 151 |  | NC_003131 | pCD1 | 70305 | NC_004088 | 4600755 | Yersinia pestis KIM | 45% | 2% | 44 | 100% |
| 154 |  | NC_003132 | pPCP1 | 9612 | NC_004088 | 4600755 | Yersinia pestis KIM | 45% | 15% | 67 | 95% |
| 203 |  | NC_004836 | pCD1 | 70504 | NC_004088 | 4600755 | Yersinia pestis KIM | 45% | 2% | 44 | 100% |
| 206 |  | NC_004837 | pPCP1 | 9610 | NC_004088 | 4600755 | Yersinia pestis KIM | 45% | 17% | 67 | 96% |
| 209 |  | NC_004839 | pCD1 | 70559 | NC_004088 | 4600755 | Yersinia pestis KIM | 45% | 2% | 44 | 100% |
| 325 |  | NC_005813 | pCD1 | 70159 | NC_004088 | 4600755 | Yersinia pestis KIM | 45% | 2% | 44 | 100% |
| 328 |  | NC_005814 | pCRY | 21742 | NC_004088 | 4600755 | Yersinia pestis KIM | 49% | 78% | 47 | 97% |
| 331 |  | NC_005816 | pPCP1 | 9609 | NC_004088 | 4600755 | Yersinia pestis KIM | 45% | 15% | 68 | 96% |

Supplementary table S3. Dinucleotide composition and GC percentage analyses of plasmid sequences with the genome

sequences of a single representative strain. Depicted are the different plasmids (accession numbers), their length, their

corresponding genome sequences (and accession number and size), the GC percentage of the plasmid, the amount of genomic

fragments with a lower GC% (ppGC), the genomic dissimilarity (δ*x1000) and the amount of genomic fragments with a lower δ*

(ppδ*).

|  | **Plasmid accession number** | **Plasmid Name** | **Plasmid size (bp)** | **Genome accession number** | **Genome size (bp)** | **Organism** | **GC%** | **ppGC** | **δ***  **(x1000)** | **ppδ*** |
| --- | --- | --- | --- | --- | --- | --- | --- | --- | --- | --- |
| 62 | NC_002146 | pX02 | 96231 | NC_005945 | 5228663 | Bacillus anthracis str. Sterne | 33% | 7% | 42 | 100% |
| 134 | NC_004721 | pBClin15 | 15274 | NC_004722 | 5411809 | Bacillus cereus ATCC 14579 | 38% | 92% | 70 | 96% |
| 192 | NC_005308 | pFL7 | 7853 | NC_006270 | 4222336 | Bacillus licheniformis ATCC 14580 | 44% | 12% | 66 | 91% |
| 193 | NC_005311 | pFL5 | 9150 | NC_006270 | 4222336 | Bacillus licheniformis ATCC 14580 | 41% | 5% | 63 | 90% |
| 20 | NC_001764 | pTA1040 | 7837 | NC_000964 | 4214630 | Bacillus subtilis subsp. subtilis str. 168 | 37% | 6% | 49 | 76% |
| 21 | NC_001765 | pTA1015 | 5807 | NC_000964 | 4214630 | Bacillus subtilis subsp. subtilis str. 168 | 41% | 15% | 71 | 89% |
| 22 | NC_001766 | pTA1060 | 8737 | NC_000964 | 4214630 | Bacillus subtilis subsp. subtilis str. 168 | 38% | 7% | 50 | 78% |
| 38 | NC_002075 | p1414 | 7949 | NC_000964 | 4214630 | Bacillus subtilis subsp. subtilis str. 168 | 38% | 8% | 74 | 91% |
| 6 | NC_001272 | pBMB9741 | 6584 | NC_005957 | 5237682 | Bacillus thuringiensis serovar konkukian str. 97-27 | 32% | 10% | 55 | 77% |
| 12 | NC_001446 | pTX14-3 | 7649 | NC_005957 | 5237682 | Bacillus thuringiensis serovar konkukian str. 97-27 | 35% | 46% | 101 | 98% |
| 118 | NC_004334 | pTX14-2 | 6829 | NC_005957 | 5237682 | Bacillus thuringiensis serovar konkukian str. 97-27 | 36% | 63% | 110 | 99% |
| 202 | NC_005567 | pGI3 | 11365 | NC_005957 | 5237682 | Bacillus thuringiensis serovar konkukian str. 97-27 | 32% | 4% | 54 | 86% |
| 230 | NC_006578 | pBT9727 | 77112 | NC_005957 | 5237682 | Bacillus thuringiensis serovar konkukian str. 97-27 | 33% | 6% | 58 | 100% |
| 184 | NC_005026 | pBI143 | 2747 | NC_006347 | 5277274 | Bacteroides fragilis YCH46 | 41% | 29% | 101 | 97% |
| 221 | NC_006297 | pBFY46 | 33716 | NC_006347 | 5277274 | Bacteroides fragilis YCH46 | 34% | 1% | 69 | 100% |
| 130 | NC_004703 | p5482 | 33038 | NC_004663 | 6260361 | Bacteroides thetaiotaomicron VPI-5482 | 47% | 98% | 94 | 100% |
| 5 | NC_000957 | lp5 | 5228 | NC_001318 | 910724 | BBUR Borrelia burgdorferi B31 | 24% | 1% | 97 | 98% |
| 31 | NC_001849 | lp17 | 16823 | NC_001318 | 910724 | BBUR Borrelia burgdorferi B31 | 23% | 2% | 69 | 100% |
| 32 | NC_001904 | cp9 | 9386 | NC_001318 | 910724 | BBUR Borrelia burgdorferi B31 | 24% | 1% | 76 | 99% |
| 78 | NC_002635 | pKJ36 | 3625 | NC_004307 | 2256646 | Bifidobacterium longum NCC2705 | 65% | 98% | 121 | 99% |
| 114 | NC_004252 | pDOJH10L | 10073 | NC_004307 | 2256646 | Bifidobacterium longum NCC2705 | 62% | 87% | 96 | 100% |
| 115 | NC_004253 | pDOJH10S | 3661 | NC_004307 | 2256646 | Bifidobacterium longum NCC2705 | 66% | 99% | 43 | 34% |
| 120 | NC_004443 | pB44 | 3624 | NC_004307 | 2256646 | Bifidobacterium longum NCC2705 | 65% | 98% | 123 | 99% |
| 138 | NC_004768 | pNAC3 | 10224 | NC_004307 | 2256646 | Bifidobacterium longum NCC2705 | 62% | 85% | 58 | 90% |
| 139 | NC_004769 | pNAC2 | 3684 | NC_004307 | 2256646 | Bifidobacterium longum NCC2705 | 65% | 96% | 126 | 99% |
| 140 | NC_004770 | pNAC1 | 3538 | NC_004307 | 2256646 | Bifidobacterium longum NCC2705 | 59% | 25% | 107 | 97% |
| 149 | NC_004943 | pBLO1 | 3626 | NC_004307 | 2256646 | Bifidobacterium longum NCC2705 | 65% | 97% | 109 | 97% |
| 162 | NC_004978 | pKJ50 | 4960 | NC_004307 | 2256646 | Bifidobacterium longum NCC2705 | 62% | 78% | 88 | 96% |
| 165 | NC_004997 | pCJ419 | 4013 | NC_003912 | 1777831 | Campylobacter jejuni RM1221 | 30% | 47% | 87 | 93% |
| 66 | NC_002182 | pMoPn | 7501 | NC_002620 | 1072950 | Chlamydia muridarum Nigg | 36% | 1% | 30 | 38% |
| 133 | NC_004720 | pCpGP1 | 7966 | NC_003361 | 1173390 | Chlamydophila caviae GPIC | 34% | 1% | 56 | 97% |
| 88 | NC_003042 | pCP13 | 54310 | NC_003366 | 3031430 | Clostridium perfringens str. 13 | 26% | 2% | 39 | 89% |
| 96 | NC_003239 | pNGA2 | 4191 | NC_002935 | 2488635 | Corynebacterium diphtheriae NCTC 13129 | 51% | 29% | 39 | 29% |
| 167 | NC_005001 | pNG2 | 15100 | NC_002935 | 2488635 | Corynebacterium diphtheriae NCTC 13129 | 56% | 83% | 38 | 72% |
| 116 | NC_004319 | pCE2 | 23743 | NC_004369 | 3147090 | Corynebacterium efficiens YS-314 | 54% | 1% | 112 | 100% |
| 117 | NC_004320 | pCE3 | 48672 | NC_004369 | 3147090 | Corynebacterium efficiens YS-314 | 56% | 2% | 105 | 100% |
| 9 | NC_001385 | pAM330 | 4448 | NC_006958 | 3282708 | Corynebacterium glutamicum ATCC 13032 | 53% | 35% | 86 | 93% |
| 11 | NC_001415 | pAG1 | 19751 | NC_006958 | 3282708 | Corynebacterium glutamicum ATCC 13032 | 59% | 100% | 84 | 99% |
| 13 | NC_001456 | pSR1 | 3054 | NC_006958 | 3282708 | Corynebacterium glutamicum ATCC 13032 | 57% | 89% | 125 | 98% |
| 26 | NC_001791 | pXZ10145.1 | 4885 | NC_006958 | 3282708 | Corynebacterium glutamicum ATCC 13032 | 60% | 99% | 71 | 87% |
| 43 | NC_002099 | pXZ10142 | 2444 | NC_006958 | 3282708 | Corynebacterium glutamicum ATCC 13032 | 57% | 89% | 109 | 95% |
| 46 | NC_002115 | ATCC 13869 native 4.45 kb plasmid | 4457 | NC_006958 | 3282708 | Corynebacterium glutamicum ATCC 13032 | 53% | 35% | 89 | 94% |
| 95 | NC_003227 | pTET3 | 27856 | NC_006958 | 3282708 | Corynebacterium glutamicum ATCC 13032 | 55% | 71% | 55 | 93% |
| 123 | NC_004533 | pAG3 | 4603 | NC_006958 | 3282708 | Corynebacterium glutamicum ATCC 13032 | 52% | 17% | 84 | 93% |
| 124 | NC_004534 | pCG2 | 6758 | NC_006958 | 3282708 | Corynebacterium glutamicum ATCC 13032 | 51% | 10% | 214 | 100% |
| 125 | NC_004535 | pGA2 | 19218 | NC_006958 | 3282708 | Corynebacterium glutamicum ATCC 13032 | 51% | 7% | 57 | 91% |
| 148 | NC_004941 | pXZ608 | 5949 | NC_006958 | 3282708 | Corynebacterium glutamicum ATCC 13032 | 56% | 85% | 164 | 100% |
| 151 | NC_004945 | pCG4 | 29371 | NC_006958 | 3282708 | Corynebacterium glutamicum ATCC 13032 | 52% | 9% | 55 | 94% |
| 47 | NC_002118 | QpH1 | 37329 | NC_002971 | 1995275 | Coxiella burnetii RSA 493 | 39% | 2% | 62 | 100% |
| 56 | NC_002131 | QpDV | 32601 | NC_002971 | 1995275 | Coxiella burnetii RSA 493 | 40% | 3% | 62 | 100% |
| 131 | NC_004704 | pQpH1 | 37393 | NC_002971 | 1995275 | Coxiella burnetii RSA 493 | 39% | 2% | 62 | 100% |
| 77 | NC_002630 | pAM373 | 36804 | NC_004668 | 3218031 | Enterococcus faecalis V583 | 35% | 7% | 45 | 92% |
| 128 | NC_004670 | pTEF3 | 17963 | NC_004668 | 3218031 | Enterococcus faecalis V583 | 33% | 4% | 72 | 97% |
| 129 | NC_004671 | pTEF2 | 57660 | NC_004668 | 3218031 | Enterococcus faecalis V583 | 34% | 4% | 54 | 95% |
| 175 | NC_005010 | pEF1071 | 9328 | NC_004668 | 3218031 | Enterococcus faecalis V583 | 37% | 40% | 53 | 82% |
| 177 | NC_005013 | pAMalpha1 | 9759 | NC_004668 | 3218031 | Enterococcus faecalis V583 | 36% | 17% | 52 | 81% |
| 48 | NC_002119 | CloDF13 | 9957 | NC_004431 | 5231428 | Escherichia coli CFT073 | 54% | 95% | 77 | 92% |
| 52 | NC_002127 | pOSAK1 | 3306 | NC_004431 | 5231428 | Escherichia coli CFT073 | 43% | 7% | 64 | 74% |
| 54 | NC_002128 | pO157 | 92721 | NC_004431 | 5231428 | Escherichia coli CFT073 | 48% | 4% | 64 | 100% |
| 59 | NC_002142 | pB171 | 68817 | NC_004431 | 5231428 | Escherichia coli CFT073 | 46% | 1% | 63 | 97% |
| 69 | NC_002482 | pGBG1 | 7620 | NC_004431 | 5231428 | Escherichia coli CFT073 | 52% | 74% | 80 | 92% |
| 70 | NC_002487 | pCol-let | 5847 | NC_004431 | 5231428 | Escherichia coli CFT073 | 47% | 13% | 77 | 90% |
| 76 | NC_002525 | R721 | 75582 | NC_004431 | 5231428 | Escherichia coli CFT073 | 43% | 1% | 57 | 99% |
| 107 | NC_003435 | pCA4 | 3078 | NC_004431 | 5231428 | Escherichia coli CFT073 | 50% | 37% | 87 | 89% |
| 179 | NC_005019 | pLG13 | 6293 | NC_004431 | 5231428 | Escherichia coli CFT073 | 44% | 4% | 97 | 96% |
| 197 | NC_005327 | pC15-1a | 92353 | NC_004431 | 5231428 | Escherichia coli CFT073 | 53% | 98% | 50 | 100% |
| 214 | NC_005923 | pFL129 | 6464 | NC_004431 | 5231428 | Escherichia coli CFT073 | 52% | 74% | 73 | 90% |
| 216 | NC_005970 | pRK2 | 5360 | NC_000913 | 4639675 | Escherichia coli K12 | 46% | 8% | 104 | 99% |
| 53 | NC_002127 | pOSAK1 | 3306 | NC_002695 | 5498450 | Escherichia coli O157:H7 | 43% | 7% | 63 | 72% |
| 61 | NC_002145 | pKL1 | 1549 | NC_002695 | 5498450 | Escherichia coli O157:H7 | 51% | 44% | 70 | 56% |
| 190 | NC_005248 | pIGAL1 | 8145 | NC_002695 | 5498450 | Escherichia coli O157:H7 | 51% | 42% | 63 | 86% |
| 15 | NC_001537 | pECO29 | 3895 | NC_002655 | 5528445 | Escherichia coli O157:H7 EDL933 | 46% | 13% | 76 | 87% |
| 119 | NC_004429 | pIS2 | 6349 | NC_002655 | 5528445 | Escherichia coli O157:H7 EDL933 | 47% | 16% | 41 | 52% |
| 196 | NC_005324 | p9123 | 6222 | NC_002655 | 5528445 | Escherichia coli O157:H7 EDL933 | 52% | 71% | 47 | 63% |
| 203 | NC_005568 | pBHRK18 | 5721 | NC_002655 | 5528445 | Escherichia coli O157:H7 EDL933 | 57% | 98% | 47 | 60% |
| 204 | NC_005569 | pBHRK19 | 5721 | NC_002655 | 5528445 | Escherichia coli O157:H7 EDL933 | 57% | 98% | 46 | 59% |
| 44 | NC_002109 | pOM1 | 4442 | NC_006570 | 1892819 | Francisella tularensis subsp. tularensis | 41% | 100% | 91 | 99% |
| 35 | NC_002002 | pPA52 | 6281 | NC_003454 | 2174500 | Fusobacterium nucleatum subsp. nucleatum ATCC 25586 | 23% | 2% | 74 | 92% |
| 89 | NC_003091 | pKH9 | 4975 | NC_003454 | 2174500 | Fusobacterium nucleatum subsp. nucleatum ATCC 25586 | 29% | 85% | 81 | 93% |
| 161 | NC_004974 | pFN1 | 5887 | NC_003454 | 2174500 | Fusobacterium nucleatum subsp. nucleatum ATCC 25586 | 23% | 3% | 77 | 95% |
| 229 | NC_006509 | pHTA426 | 47890 | NC_006510 | 3544776 | Geobacillus kaustophilus HTA426 | 44% | 1% | 123 | 100% |
| 105 | NC_003374 | pGO128 | 4340 | NC_006677 | 2702173 | Gluconobacter oxydans 621H | 52% | 2% | 128 | 100% |
| 122 | NC_004458 | pAG5 | 5648 | NC_006677 | 2702173 | Gluconobacter oxydans 621H | 51% | 1% | 85 | 98% |
| 198 | NC_005329 | pNAD1 | 5568 | NC_002940 | 1698955 | Haemophilus ducreyi 35000HP | 34% | 3% | 66 | 86% |
| 49 | NC_002121 | pHSB | 1736 | NC_002607 | 2014239 | Halobacterium salinarum NRC-1 | 64% | 12% | 79 | 60% |
| 94 | NC_003158 | pHH205 | 16341 | NC_002607 | 2014239 | Halobacterium salinarum NRC-1 | 61% | 4% | 113 | 100% |
| 18 | NC_001756 | pHPM180 | 3506 | NC_000915 | 1667867 | Helicobacter pylori 26695 | 37% | 19% | 104 | 95% |
| 29 | NC_001843 | pHP489 | 1222 | NC_000915 | 1667867 | Helicobacter pylori 26695 | 33% | 6% | 158 | 97% |
| 45 | NC_002110 | pHPO100 | 3520 | NC_000915 | 1667867 | Helicobacter pylori 26695 | 36% | 11% | 105 | 96% |
| 137 | NC_004767 | pHP51 | 3955 | NC_000915 | 1667867 | Helicobacter pylori 26695 | 35% | 7% | 90 | 95% |
| 146 | NC_004845 | pHPM8 | 7817 | NC_000915 | 1667867 | Helicobacter pylori 26695 | 33% | 3% | 120 | 99% |
| 153 | NC_004950 | pHel4 | 10970 | NC_000915 | 1667867 | Helicobacter pylori 26695 | 34% | 5% | 138 | 100% |
| 213 | NC_005917 | pAL202 | 12120 | NC_000915 | 1667867 | Helicobacter pylori 26695 | 34% | 4% | 147 | 100% |
| 14 | NC_001476 | pHPM186 | 12887 | NC_000921 | 1643831 | Helicobacter pylori, strain J99 complete genome | 36% | 3% | 107 | 98% |
| 152 | NC_004949 | Helicobacter pHel5 | 18291 | NC_000921 | 1643831 | Helicobacter pylori, strain J99 complete genome | 34% | 1% | 140 | 100% |
| 108 | NC_003458 | pLA103 | 13913 | NC_006814 | 1993564 | Lactobacillus acidophilus NCFM | 40% | 99% | 60 | 92% |
| 164 | NC_004985 | pLA106 | 2862 | NC_006814 | 1993564 | Lactobacillus acidophilus NCFM | 43% | 99% | 78 | 82% |
| 50 | NC_002123 | pLTK2 | 2295 | NC_004567 | 3308274 | Lactobacillus plantarum WCFS1 | 39% | 6% | 119 | 98% |
| 109 | NC_003893 | pLP2000 | 2061 | NC_004567 | 3308274 | Lactobacillus plantarum WCFS1 | 38% | 5% | 123 | 98% |
| 110 | NC_003894 | pLP9000 | 9253 | NC_004567 | 3308274 | Lactobacillus plantarum WCFS1 | 37% | 2% | 75 | 96% |
| 150 | NC_004944 | pMD5057 | 10877 | NC_004567 | 3308274 | Lactobacillus plantarum WCFS1 | 36% | 0% | 83 | 99% |
| 220 | NC_006278 | p256 | 7222 | NC_004567 | 3308274 | Lactobacillus plantarum WCFS1 | 37% | 2% | 86 | 97% |
| 224 | NC_006375 | pWCFS101 | 1917 | NC_004567 | 3308274 | Lactobacillus plantarum WCFS1 | 39% | 8% | 54 | 45% |
| 225 | NC_006376 | pWCFS102 | 2365 | NC_004567 | 3308274 | Lactobacillus plantarum WCFS1 | 34% | 1% | 122 | 98% |
| 226 | NC_006377 | pWCFS103 | 36069 | NC_004567 | 3308274 | Lactobacillus plantarum WCFS1 | 41% | 5% | 70 | 100% |
| 227 | NC_006399 | pPB1 | 2899 | NC_004567 | 3308274 | Lactobacillus plantarum WCFS1 | 38% | 4% | 110 | 98% |
| 2 | NC_000906 | pIL105 | 8506 | NC_002662 | 2365589 | Lactococcus lactis subsp. lactis Il1403 | 30% | 0% | 55 | 78% |
| 58 | NC_002138 | pCIS3 | 6159 | NC_002662 | 2365589 | Lactococcus lactis subsp. lactis Il1403 | 36% | 65% | 47 | 57% |
| 63 | NC_002150 | pAH33 | 6159 | NC_002662 | 2365589 | Lactococcus lactis subsp. lactis Il1403 | 36% | 65% | 47 | 57% |
| 67 | NC_002192 | pWV01 | 2178 | NC_002662 | 2365589 | Lactococcus lactis subsp. lactis Il1403 | 33% | 22% | 140 | 98% |
| 68 | NC_002193 | pWVO2 | 3826 | NC_002662 | 2365589 | Lactococcus lactis subsp. lactis Il1403 | 31% | 3% | 164 | 100% |
| 73 | NC_002502 | pCI305 | 8694 | NC_002662 | 2365589 | Lactococcus lactis subsp. lactis Il1403 | 32% | 5% | 59 | 83% |
| 85 | NC_002798 | pSRQ700 | 7784 | NC_002662 | 2365589 | Lactococcus lactis subsp. lactis Il1403 | 34% | 22% | 62 | 83% |
| 86 | NC_002799 | pCRL291.1 | 4640 | NC_002662 | 2365589 | Lactococcus lactis subsp. lactis Il1403 | 34% | 17% | 108 | 97% |
| 90 | NC_003101 | pCRL1127 | 8278 | NC_002662 | 2365589 | Lactococcus lactis subsp. lactis Il1403 | 35% | 39% | 59 | 80% |
| 147 | NC_004922 | pMN5 | 5670 | NC_002662 | 2365589 | Lactococcus lactis subsp. lactis Il1403 | 30% | 1% | 51 | 61% |
| 154 | NC_004955 | pBL1 | 10899 | NC_002662 | 2365589 | Lactococcus lactis subsp. lactis Il1403 | 33% | 6% | 72 | 94% |
| 156 | NC_004959 | pSRQ900 | 10836 | NC_002662 | 2365589 | Lactococcus lactis subsp. lactis Il1403 | 31% | 1% | 68 | 92% |
| 157 | NC_004960 | pSRQ800 | 7858 | NC_002662 | 2365589 | Lactococcus lactis subsp. lactis Il1403 | 31% | 2% | 45 | 63% |
| 158 | NC_004966 | pAH82 | 20331 | NC_002662 | 2365589 | Lactococcus lactis subsp. lactis Il1403 | 34% | 22% | 52 | 94% |
| 163 | NC_004981 | pCL2.1 | 2112 | NC_002662 | 2365589 | Lactococcus lactis subsp. lactis Il1403 | 34% | 27% | 69 | 54% |
| 223 | NC_006366 | pLPL | 59832 | NC_006369 | 3345687 | Legionella pneumophila str. Lens | 38% | 45% | 27 | 96% |
| 17 | NC_001733 | small extrachromosomal element | 16550 | NC_000909 | 1664970 | Methanocaldococcus jannaschii DSM 2661 | 29% | 3% | 62 | 93% |
| 28 | NC_001811 | pURB500 | 8285 | NC_005791 | 1661137 | Methanococcus maripaludis S2 | 27% | 1% | 105 | 98% |
| 41 | NC_002097 | pC2A | 5467 | NC_003552 | 5751492 | Methanosarcina acetivorans C2A | 38% | 10% | 55 | 69% |
| 1 | NC_000905 | pME2200 | 6205 | NC_000916 | 1751377 | Methanothermobacter thermautotrophicus str. Delta H | 45% | 9% | 108 | 98% |
| 7 | NC_001336 | plasmid pFV1 | 13514 | NC_000916 | 1751377 | Methanothermobacter thermautotrophicus str. Delta H | 42% | 3% | 127 | 100% |
| 8 | NC_001337 | plasmid pZF1 | 11014 | NC_000916 | 1751377 | Methanothermobacter thermautotrophicus str. Delta H | 43% | 3% | 117 | 99% |
| 51 | NC_002125 | pME2001 | 4439 | NC_000916 | 1751377 | Methanothermobacter thermautotrophicus str. Delta H | 45% | 9% | 69 | 89% |
| 178 | NC_005016 | pVT2 | 12868 | NC_002944 | 4829781 | Mycobacterium avium subsp. paratuberculosis str. k10 | 66% | 8% | 56 | 99% |
| 42 | NC_002098 | pJD4 | 7426 | NC_002946 | 2153922 | Neisseria gonorrhoeae FA 1090 | 38% | 0% | 117 | 99% |
| 136 | NC_004758 | pJS-B | 7245 | NC_003112 | 2272351 | Neisseria meningitidis MC58 | 42% | 3% | 86 | 91% |
| 222 | NC_006363 | pNF2 | 87093 | NC_006361 | 6021225 | Nocardia farcinica IFM 10152 | 68% | 3% | 33 | 100% |
| 97 | NC_003241 | pCC7120zeta | 5584 | NC_003272 | 6413771 | Nostoc sp. PCC 7120 | 44% | 91% | 76 | 97% |
| 99 | NC_003267 | pCC7120gamma | 101965 | NC_003272 | 6413771 | Nostoc sp. PCC 7120 | 41% | 29% | 40 | 100% |
| 100 | NC_003270 | pCC7120epsilon | 40340 | NC_003272 | 6413771 | Nostoc sp. PCC 7120 | 41% | 30% | 22 | 82% |
| 101 | NC_003273 | pCC7120delta | 55414 | NC_003272 | 6413771 | Nostoc sp. PCC 7120 | 42% | 64% | 33 | 99% |
| 25 | NC_001774 | pIG1 | 5360 | NC_002663 | 2257487 | Pasteurella multocida subsp. multocida str. Pm70 | 47% | 100% | 84 | 94% |
| 141 | NC_004771 | pJR1 | 6792 | NC_002663 | 2257487 | Pasteurella multocida subsp. multocida str. Pm70 | 46% | 99% | 89 | 96% |
| 142 | NC_004772 | pJR2 | 5252 | NC_002663 | 2257487 | Pasteurella multocida subsp. multocida str. Pm70 | 42% | 77% | 59 | 86% |
| 75 | NC_002518 | pPP81 | 2534 | NC_002947 | 6181863 | Pseudomonas putida KT2440 | 59% | 16% | 96 | 91% |
| 104 | NC_003350 | pWW0 | 116580 | NC_002947 | 6181863 | Pseudomonas putida KT2440 | 59% | 4% | 47 | 100% |
| 166 | NC_004999 | pDTG1 | 83042 | NC_002947 | 6181863 | Pseudomonas putida KT2440 | 56% | 4% | 51 | 99% |
| 174 | NC_005009 | pYQ39 | 2297 | NC_002947 | 6181863 | Pseudomonas putida KT2440 | 59% | 16% | 62 | 53% |
| 24 | NC_001773 | pGT5 | 3444 | NC_000868 | 1765118 | Pyrococcus abyssi GE5 | 43% | 29% | 63 | 82% |
| 10 | NC_001399 | pJTPS1 | 6633 | NC_003295 | 3716413 | Ralstonia solanacearum GMI1000 | 62% | 8% | 42 | 44% |
| 191 | NC_005297 | pRPA | 8427 | NC_005296 | 5459213 | Rhodopseudomonas palustris CGA009 | 60% | 2% | 160 | 100% |
| 79 | NC_002638 | pKDSC50 | 49503 | NC_003198 | 4809037 | Salmonella enterica subsp. enterica serovar Typhi str. CT18 | 52% | 40% | 95 | 100% |
| 210 | NC_005862 | cryptic plasmid | 6066 | NC_004631 | 4791961 | Salmonella enterica subsp. enterica serovar Typhi Ty2 | 54% | 62% | 60 | 81% |
| 37 | NC_002056 | pSC101 | 9263 | NC_003197 | 4857432 | Salmonella typhimurium LT2 | 51% | 28% | 76 | 95% |
| 102 | NC_003277 | pSLT | 93939 | NC_003197 | 4857432 | Salmonella typhimurium LT2 | 53% | 80% | 95 | 100% |
| 82 | NC_002773 | p2457TS2 | 3179 | NC_004741 | 4599354 | Shigella flexneri 2a str. 2457T | 45% | 5% | 62 | 77% |
| 74 | NC_002517 | p21 | 20719 | NC_002951 | 2809422 | Staphylococcus aureus subsp. aureus COL | 29% | 1% | 82 | 97% |
| 19 | NC_001763 | J3358 | 6024 | NC_002952 | 2902619 | Staphylococcus aureus subsp. aureus MRSA252 | 31% | 19% | 68 | 83% |
| 23 | NC_001767 | pKH6 | 4439 | NC_002952 | 2902619 | Staphylococcus aureus subsp. aureus MRSA252 | 30% | 9% | 80 | 86% |
| 36 | NC_002013 | pC194 | 2910 | NC_002952 | 2902619 | Staphylococcus aureus subsp. aureus MRSA252 | 29% | 8% | 124 | 96% |
| 55 | NC_002129 | pC221 | 4557 | NC_002952 | 2902619 | Staphylococcus aureus subsp. aureus MRSA252 | 32% | 29% | 61 | 73% |
| 64 | NC_002156 | J3356::POX7;1 | 8007 | NC_002952 | 2902619 | Staphylococcus aureus subsp. aureus MRSA252 | 39% | 99% | 70 | 89% |
| 98 | NC_003265 | pETB | 38211 | NC_002952 | 2902619 | Staphylococcus aureus subsp. aureus MRSA252 | 28% | 1% | 91 | 100% |
| 185 | NC_005054 | pLW043 | 57889 | NC_002952 | 2902619 | Staphylococcus aureus subsp. aureus MRSA252 | 31% | 6% | 73 | 100% |
| 189 | NC_005243 | pC223 | 4608 | NC_002952 | 2902619 | Staphylococcus aureus subsp. aureus MRSA252 | 31% | 25% | 82 | 89% |
| 199 | NC_005564 | pS194 | 4397 | NC_002952 | 2902619 | Staphylococcus aureus subsp. aureus MRSA252 | 32% | 27% | 70 | 80% |
| 212 | NC_005908 | pE194 | 3728 | NC_002952 | 2902619 | Staphylococcus aureus subsp. aureus MRSA252 | 32% | 31% | 90 | 91% |
| 16 | NC_001565 | pUB110 | 2266 | NC_002758 | 2878529 | Staphylococcus aureus subsp. aureus Mu50 | 36% | 88% | 89 | 81% |
| 40 | NC_002096 | pKH7 | 4118 | NC_002758 | 2878529 | Staphylococcus aureus subsp. aureus Mu50 | 29% | 3% | 61 | 68% |
| 65 | NC_002157 | J3356::pOX7;3 | 8007 | NC_002758 | 2878529 | Staphylococcus aureus subsp. aureus Mu50 | 38% | 98% | 70 | 89% |
| 83 | NC_002774 | pVRSAp | 25107 | NC_002758 | 2878529 | Staphylococcus aureus subsp. aureus Mu50 | 29% | 1% | 69 | 96% |
| 93 | NC_003140 | pN315 | 24653 | NC_002758 | 2878529 | Staphylococcus aureus subsp. aureus Mu50 | 29% | 1% | 78 | 97% |
| 127 | NC_004562 | pNVH01 | 2650 | NC_002758 | 2878529 | Staphylococcus aureus subsp. aureus Mu50 | 30% | 14% | 95 | 88% |
| 176 | NC_005011 | pMW2 | 20654 | NC_002758 | 2878529 | Staphylococcus aureus subsp. aureus Mu50 | 28% | 1% | 80 | 96% |
| 183 | NC_005024 | pSK41 | 46445 | NC_002758 | 2878529 | Staphylococcus aureus subsp. aureus Mu50 | 30% | 2% | 74 | 100% |
| 187 | NC_005127 | pUB101 | 21845 | NC_002758 | 2878529 | Staphylococcus aureus subsp. aureus Mu50 | 29% | 1% | 69 | 94% |
| 215 | NC_005951 | Staphylococcus pSAS | 20652 | NC_002758 | 2878529 | Staphylococcus aureus subsp. aureus Mu50 | 28% | 1% | 80 | 97% |
| 180 | NC_005020 | pKH3 | 2979 | NC_003923 | 2820462 | Staphylococcus aureus subsp. aureus MW2 | 29% | 6% | 103 | 92% |
| 200 | NC_005565 | pSN2 | 1288 | NC_003923 | 2820462 | Staphylococcus aureus subsp. aureus MW2 | 36% | 90% | 134 | 92% |
| 34 | NC_001995 | pSK6 | 1551 | NC_002745 | 2814816 | Staphylococcus aureus subsp. aureus N315 | 34% | 62% | 118 | 90% |
| 113 | NC_003969 | pSepCH | 2391 | NC_004461 | 2499279 | Staphylococcus epidermidis ATCC 12228 | 29% | 12% | 146 | 98% |
| 171 | NC_005006 | pSE-12228-03 | 8007 | NC_004461 | 2499279 | Staphylococcus epidermidis ATCC 12228 | 36% | 96% | 121 | 99% |
| 201 | NC_005566 | pSK639 | 8013 | NC_004461 | 2499279 | Staphylococcus epidermidis ATCC 12228 | 34% | 87% | 48 | 77% |
| 168 | NC_005003 | pSE-12228-06 | 6585 | NC_002976 | 2616530 | Staphylococcus epidermidis RP62A | 32% | 36% | 91 | 95% |
| 169 | NC_005004 | pSE-12228-05 | 24365 | NC_002976 | 2616530 | Staphylococcus epidermidis RP62A | 29% | 4% | 44 | 87% |
| 170 | NC_005005 | pSE-12228-04 | 17261 | NC_002976 | 2616530 | Staphylococcus epidermidis RP62A | 28% | 1% | 43 | 82% |
| 172 | NC_005007 | pSE-12228-02 | 4679 | NC_002976 | 2616530 | Staphylococcus epidermidis RP62A | 32% | 44% | 60 | 75% |
| 173 | NC_005008 | pSE-12228-01 | 4439 | NC_002976 | 2616530 | Staphylococcus epidermidis RP62A | 30% | 14% | 61 | 74% |
| 27 | NC_001797 | pGB354 | 6437 | NC_004368 | 2211485 | Streptococcus agalactiae NEM316 | 33% | 7% | 97 | 98% |
| 57 | NC_002136 | pGB3631 | 5842 | NC_004368 | 2211485 | Streptococcus agalactiae NEM316 | 34% | 13% | 97 | 97% |
| 81 | NC_002757 | pUA140 | 5640 | NC_004350 | 2030921 | Streptococcus mutans UA159 | 33% | 5% | 58 | 86% |
| 87 | NC_002810 | pLM7 | 5658 | NC_004350 | 2030921 | Streptococcus mutans UA159 | 33% | 5% | 59 | 89% |
| 181 | NC_005021 | pSMB1 | 3162 | NC_003098 | 2038615 | Streptococcus pneumoniae R6 complete genome | 32% | 5% | 86 | 92% |
| 182 | NC_005022 | pDP1 | 3161 | NC_003028 | 2160837 | Streptococcus pneumoniae TIGR4 | 32% | 4% | 87 | 91% |
| 217 | NC_006130 | pDN571 | 3351 | NC_003485 | 1895017 | Streptococcus pyogenes strain MGAS8232 | 34% | 6% | 51 | 47% |
| 3 | NC_000937 | pER35 | 9531 | NC_006449 | 1796226 | Streptococcus thermophilus CNRZ1066 | 37% | 10% | 89 | 99% |
| 84 | NC_002776 | pER13 | 4139 | NC_006449 | 1796226 | Streptococcus thermophilus CNRZ1066 | 38% | 36% | 74 | 90% |
| 160 | NC_004968 | pER371 | 2672 | NC_006449 | 1796226 | Streptococcus thermophilus CNRZ1066 | 38% | 32% | 115 | 97% |
| 194 | NC_005322 | pSMQ308 | 8144 | NC_006449 | 1796226 | Streptococcus thermophilus CNRZ1066 | 38% | 24% | 173 | 100% |
| 195 | NC_005323 | pSMQ173b | 4449 | NC_006449 | 1796226 | Streptococcus thermophilus CNRZ1066 | 37% | 15% | 85 | 94% |
| 4 | NC_000938 | pER36 | 3498 | NC_006448 | 1796846 | Streptococcus thermophilus LMG 18311 | 34% | 6% | 77 | 88% |
| 135 | NC_004747 | pND103 | 3531 | NC_006448 | 1796846 | Streptococcus thermophilus LMG 18311 | 32% | 3% | 123 | 99% |
| 155 | NC_004958 | pSMQ172 | 4230 | NC_006448 | 1796846 | Streptococcus thermophilus LMG 18311 | 38% | 29% | 80 | 91% |
| 186 | NC_005098 | pt38 | 2911 | NC_006448 | 1796846 | Streptococcus thermophilus LMG 18311 | 32% | 3% | 72 | 81% |
| 132 | NC_004719 | SAP1 | 94287 | NC_003155 | 9025608 | Streptomyces avermitilis MA-4680 | 69% | 11% | 53 | 99% |
| 103 | NC_003319 | p2 SCP2* | 7686 | NC_003888 | 8667507 | Streptomyces coelicolor A3(2) | 73% | 56% | 69 | 94% |
| 211 | NC_005907 | pIT3 | 4967 | NC_002754 | 2992245 | Sulfolobus solfataricus P2 | 44% | 99% | 65 | 82% |
| 33 | NC_001974 | pMA4 | 2514 | NC_005070 | 2434428 | Synechococcus sp. WH 8102 | 49% | 8% | 201 | 100% |
| 159 | NC_004967 | pCB2.4 | 2345 | NC_000911 | 3573470 | Synechocystis sp. PCC 6803 | 43% | 10% | 80 | 89% |
| 188 | NC_005231 | pSYSG | 44343 | NC_000911 | 3573470 | Synechocystis sp. PCC 6803 | 49% | 75% | 118 | 100% |
| 206 | NC_005792 | pTT8 | 9328 | NC_006461 | 1849742 | Thermus thermophilus HB8 | 69% | 32% | 65 | 87% |
| 228 | NC_006463 | pTT8 | 9322 | NC_006461 | 1849742 | Thermus thermophilus HB8 | 69% | 35% | 65 | 87% |
| 80 | NC_002650 | pTS1 | 3649 | NC_002967 | 2843201 | Treponema denticola ATCC 35405 | 34% | 15% | 60 | 56% |
| 106 | NC_003425 | pWb1 | 5280 | NC_004344 | 697724 | Wigglesworthia glossinidia endosymbiont of Glossina brevipalpis | 22% | 46% | 89 | 90% |
| 111 | NC_003921 | pXAC33 | 33700 | NC_003919 | 5175554 | Xanthomonas axonopodis pv. citri str. 306 | 62% | 5% | 64 | 95% |
| 112 | NC_003922 | pXAC64 | 64920 | NC_003919 | 5175554 | Xanthomonas axonopodis pv. citri str. 306 | 61% | 5% | 63 | 99% |
| 71 | NC_002489 | pXF1.3 | 1286 | NC_002488 | 2679306 | Xylella fastidiosa 9a5c | 56% | 74% | 99 | 78% |
| 72 | NC_002490 | pXF51 | 51158 | NC_002488 | 2679306 | Xylella fastidiosa 9a5c | 50% | 17% | 80 | 100% |
| 39 | NC_002092 | pXF868 | 1296 | NC_004556 | 2519802 | Xylella fastidiosa Temecula1 | 55% | 74% | 109 | 87% |
| 126 | NC_004554 | pXFPD1.3 | 1346 | NC_004556 | 2519802 | Xylella fastidiosa Temecula1 | 54% | 66% | 103 | 84% |
| 208 | NC_005814 | pCRY | 21742 | NC_005810 | 4595065 | Yersinia pestis biovar Medievalis str. 91001 | 49% | 79% | 47 | 97% |
| 91 | NC_003131 | pCD1 | 70305 | NC_003143 | 4653728 | Yersinia pestis CO92 | 45% | 2% | 44 | 100% |
| 143 | NC_004836 | pCD1 | 70504 | NC_003143 | 4653728 | Yersinia pestis CO92 | 45% | 2% | 44 | 100% |
| 144 | NC_004837 | pPCP1 | 9610 | NC_003143 | 4653728 | Yersinia pestis CO92 | 45% | 16% | 67 | 96% |
| 207 | NC_005813 | pCD1 | 70159 | NC_003143 | 4653728 | Yersinia pestis CO92 | 45% | 2% | 44 | 100% |
| 209 | NC_005816 | pPCP1 | 9609 | NC_003143 | 4653728 | Yersinia pestis CO92 | 45% | 17% | 67 | 96% |
| 60 | NC_002144 | pYC | 5919 | NC_004088 | 4600755 | Yersinia pestis KIM | 40% | 2% | 81 | 96% |
| 92 | NC_003132 | pPCP1 | 9612 | NC_004088 | 4600755 | Yersinia pestis KIM | 45% | 15% | 67 | 95% |
| 145 | NC_004839 | pCD1 | 70559 | NC_004088 | 4600755 | Yersinia pestis KIM | 45% | 2% | 44 | 100% |
| 218 | NC_006153 | pYV | 68526 | NC_006155 | 4744671 | Yersinia pseudotuberculosis IP 32953 | 45% | 1% | 46 | 100% |
| 219 | NC_006154 | pYptb32953 | 27702 | NC_006155 | 4744671 | Yersinia pseudotuberculosis IP 32953 | 45% | 6% | 44 | 93% |
| 30 | NC_001845 | pZMO1 | 1680 | NC_006526 | 2056416 | Zymomonas mobilis subsp. mobilis ZM4 | 39% | 3% | 87 | 82% |
| 121 | NC_004457 | p1 | 40991 | NC_006526 | 2056416 | Zymomonas mobilis subsp. mobilis ZM4 | 43% | 2% | 55 | 98% |
| 205 | NC_005701 | pZMO2 | 1669 | NC_006526 | 2056416 | Zymomonas mobilis subsp. mobilis ZM4 | 39% | 3% | 77 | 72% |
